# Giant deformation potential induced small polaron effect in Dion–Jacobson two-dimensional lead halide perovskites

**DOI:** 10.1093/nsr/nwae461

**Published:** 2024-12-16

**Authors:** Yuling Huang, Shaokuan Gong, Qianxia Chen, Congcong Chen, Zhangqiang Yang, Kang Wang, Jie Xue, Daozeng Wang, Haipeng Lu, Lingling Mao, Ye Yang, Jin-Zhu Zhao, Xihan Chen

**Affiliations:** SUSTech Energy Institute for Carbon Neutrality, Department of Mechanical and Energy Engineering, Southern University of Science and Technology, Shenzhen 518055, China; SUSTech Energy Institute for Carbon Neutrality, Department of Mechanical and Energy Engineering, Southern University of Science and Technology, Shenzhen 518055, China; Guangdong Basic Research Centre of Excellence for Structure and Fundamental Interactions of Matter, Guangdong Provincial Key Laboratory of Quantum Engineering and Quantum Materials, School of Physics, South China Normal University, Guangzhou 510006, China; Guangdong-Hong Kong Joint Laboratory of Quantum Matter, Frontier Research Institute for Physics, South China Normal University, Guangzhou 510006, China; Department of Chemistry, Southern University of Science and Technology, Shenzhen 518055, China; Department of Chemistry, Xiamen University, Xiamen 361005, China; Department of Chemistry, Xiamen University, Xiamen 361005, China; Department of Chemistry, The Hong Kong University of Science and Technology, Hong Kong 999077, China; SUSTech Energy Institute for Carbon Neutrality, Department of Mechanical and Energy Engineering, Southern University of Science and Technology, Shenzhen 518055, China; Department of Chemistry, The Hong Kong University of Science and Technology, Hong Kong 999077, China; Department of Chemistry, Southern University of Science and Technology, Shenzhen 518055, China; Department of Chemistry, Xiamen University, Xiamen 361005, China; Guangdong Basic Research Centre of Excellence for Structure and Fundamental Interactions of Matter, Guangdong Provincial Key Laboratory of Quantum Engineering and Quantum Materials, School of Physics, South China Normal University, Guangzhou 510006, China; Guangdong-Hong Kong Joint Laboratory of Quantum Matter, Frontier Research Institute for Physics, South China Normal University, Guangzhou 510006, China; Centre for Computational Science and Engineering, Southern University of Science and Technology, Shenzhen 518055, China; National Laboratory of Solid State Microstructures, Nanjing University, Nanjing 210093, China; SUSTech Energy Institute for Carbon Neutrality, Department of Mechanical and Energy Engineering, Southern University of Science and Technology, Shenzhen 518055, China

**Keywords:** ultrafast spectroscopy, coherent acoustic phonon, deformation potential, spin polarization, polaronic carriers

## Abstract

Halide perovskites have attracted substantial attention recently. However, the strong lattice distortion effects in these materials have led to debates regarding the nature of charge carriers. While the behavior of carriers in bulk three-dimensional materials is well-documented, the characteristics of carriers in two-dimensional perovskites remain less well understood. In this study, we provide direct and clear evidence of small polaron formation through transient spectroscopic analysis of deformation potential and dynamic lattice screening. Coherent acoustic phonon wave signals reveal a strong coupling between carriers and lattice degrees of freedom, leading to small polaron formation and a spin lifetime enhancement of up to 10-fold. Utilizing optical Kerr spectroscopy and theoretical modeling, we observed a notably long polarization response time at room temperature, attributed to lattice distortion and small polarons approximately two-unit cells in size. Temperature-dependent coherent phonon dynamics and X-ray diffraction further confirmed the presence of small polarons. This discovery underscores the significance of the cooperative interplay between exciton dynamics and the small polaron field, particularly in influencing the Coulomb exchange interaction of excitons.

## Teaser

A giant deformation potential in Dion-Jacobson type 2-dimensional hybrid halide perovskites induced small polaron formation revealed by elongate spin polarized lifetime.

## INTRODUCTION

Halide perovskites have emerged as highly promising materials for optoelectronic applications due to their efficient carrier transport, long carrier lifetime and defect tolerance [1–4]. However, their inherently soft and polar structure, characterized by strong electron-phonon coupling and dynamic distortion effects, makes their optical and electronic properties difficult to predict, leading to debates about the nature of carriers in perovskites [5,6]. Various models, including exciton, polaron and free carrier models, have been proposed, each with vastly different transport and recombination rates. For example, free carrier large polarons have been suggested as the primary energy carriers in methylammonium lead iodide (MAPbI_3_)-based three-dimensional (3D) perovskites [7,8]. Therefore, a clear understanding of carrier nature and its relationship with the perovskite structure is essential for designing next-generation high-performance optoelectronic devices.

Two-dimensional (2D) perovskites, with systematically tuneable organic cations, have gained significant interest for potential applications [9]. Examples such as coherent quantum beating [10], elongated spin relaxation [11,12], strong self-trapping effects [13] and phonon coherence [14] have been demonstrated. Excitons are often proposed as the primary carriers, similar to epitaxial semiconductor quantum wells. However, the peculiar nature of excitons in these structurally complex materials remains unclear. They are thought to exist in an intermediate regime between extended Wannier excitons in quantum-confined semiconductors and localized excitons in molecular semiconductors, or even polarons [5,15–17]. Understanding the true nature of energy carriers in 2D perovskites is crucial for advancing device design. Previous studies have suggested that polaronic characteristics influence the excitonic spectral structure in 2D perovskites through Raman scattering [18], exciton spin relaxation [11] and X-ray diffraction (XRD) [14]. However, these techniques only qualitatively characterize polaron formation and do not precisely distinguish between small and large polarons or elucidate their relationship with the lattice. Additionally, the role of small polarons in 2D perovskites is not fully understood. For instance, earlier studies on spin relaxation focused on decoherence mechanisms without considering polaron–lattice interactions [19], and charge carrier transport analyses and spin-resolved transient absorption (TA) spectroscopy did not directly address phonons [11]. Apart from the properties discussed above, Dion-Jacobson (DJ) phase 2D perovskites have recently been seen in many of the high-performance optoelectronic devices [20]. Compared with 2D Ruddlesden–Popper (RP) hybrid perovskites featuring monovalent cations, DJ perovskites exhibit shorter interlayer distances due to stronger interaction of diammonium cations (+2) with the inorganic layers, facilitated by both hydrogen bonding and interlayer van der Waals interactions. And these types of mixed interactions make it even harder to understand the photodynamics in DJ phase perovskite.

Therefore, in our study, we combine carrier spin measurements, the optical Kerr effect and coherent acoustic wave analysis with theoretical approaches to provide direct evidence for small polaron formation in DJ phase 2D perovskite (4AMP) (MA)*_n_*_−1_Pb*_n_*I_3_*_n_*_+1_ (*n* = 1–3), where 4AMP represents 4-(aminomethyl)piperidinium and MA represents methylammonium. Our analysis of deformation potential and dynamic lattice screening reveals a strong coupling of carriers to lattice degrees of freedom, indicated by a giant deformation potential of −123 eV, which is 30 times larger than that of conventional 2D or 3D perovskites. The formation of small polarons results in up to a 10-fold enhancement of spin lifetime for perovskites with the same binding energy. Using optical Kerr spectroscopy and theoretical modeling, we observed the longest polarization response time of nearly 600 ps at room temperature, originating from large lattice distortion, specifically small polarons approximately two-unit cells in size. Temperature-dependent coherent phonon dynamics and XRD further confirmed the formation of small polarons. Because small polarons affect the Coulomb exchange interaction of excitons, this enhancement underscores the significance of the cooperative interplay between exciton dynamics and the small polaron field.

## RESULTS

### Anomalous spin lifetime enhancement in high binding energy perovskites

**Figure 1.**
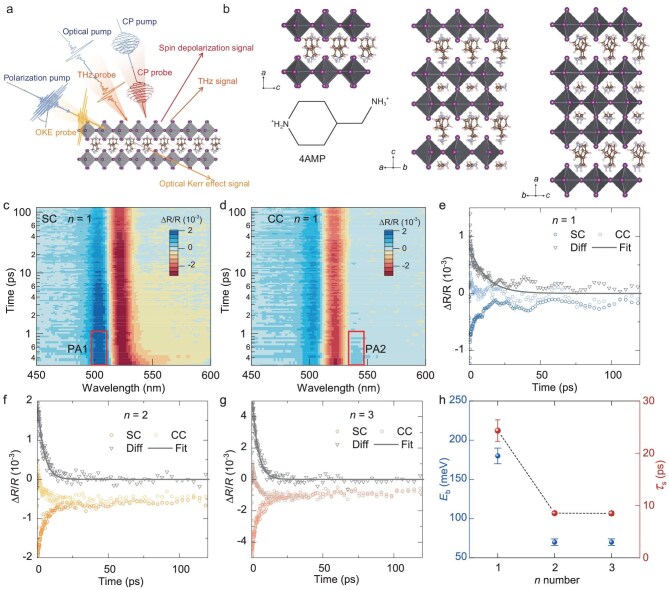
(a) Schematic illustration of small polaron characterization experiments (CP in panel a stands for circular polarized). (b) Crystal structures of (4AMP) (MA)*_n_*_−1_Pb*_n_*I_3_*_n_*_+1_ (*n* = 1, 2 and 3) series samples are reported here, where 4AMP is 4-(aminomethyl)piperidinium. Pseudo color maps of TR spectra of *n* = 1 sample with (c) same circularly polarized pump and probe pulses (*ϕ*^−^ and *ϕ*^−^) and (d) counter circularly polarized pump and probe pulses (*ϕ*  ^+^ and *ϕ*^−^). Spin relaxation dynamics of (e) *n* = 1, (f) *n* = 2 and (g) *n* = 3, where Diff denotes the net spin contribution obtained from SC–CC signals, and Fit denotes the fitted curves for spin-relaxation time ${{\tau }_s}$ using a single exponential decay model. (h) Exciton binding energy and spin lifetime of *n* = 1–3 samples.

Figure 1a presents a schematic of the experiment, in which our samples are photo-excited by an ultrafast pump pulse, with details provided in the Materials and Methods section. Figure 1b illustrates the crystal structures of various phases of DJ 2D lead iodide perovskites, (4AMP) (MA)*_n_*_−1_Pb*_n_*I_3_*_n_*_+1_ (*n* = 1, 2 and 3), where *n* denotes the number of PbI_6_ layers. The structural and optical properties characterization, analyzed using XRD, transient spectra and ultraviolet-visible (UV-vis) spectra, are displayed in Fig. S1 (Supplementary data), confirming the formation of *n* = 1–3 composites in this study. Details on exciton absorption peak, recombination dynamics and spectral evolution of spin measurement are provided in Figs S2–S7. The longest total carrier lifetime was obtained in the *n* = 1 sample, which is quite different from normal RP phase perovskites in the literature [21]. With the total carrier lifetime established, we then turned to investigate the spin-polarized lifetime, which could reflect more information on the fundamental lattice properties. Using a circularly polarized pump, pure spin states could be excited and the evolution of a spin-polarized population with time can be recorded. The general spin-dephasing mechanism consists of two parts, spin-orbit coupling (SOC)-based relaxation (D'yakonov–Perel (DP) mechanism or other SOC) and carrier phonon/impurity scattering-based relaxation (Elliott–Yafet (EY) scattering) [22]. The general rule could be written as:


(1) \begin{eqnarray*}
\frac{1}{{{{\tau }_{\mathrm{s}}}}} = \frac{1}{{{{\tau }_{{\mathrm{SOC}}}}}} + \frac{1}{{{{\tau }_{{\mathrm{scattering}}}}}},
\end{eqnarray*}

where $\frac{1}{{{{\tau }_{\mathrm{s}}}}}$ represents the total relaxation rate, $\frac{1}{{{{\tau }_{{\mathrm{SOC}}}}}}$ represents the SOC-based relaxation rate and the $\frac{1}{{{{\tau }_{{\mathrm{scattering}}}}}}$ represents the scattering rate of carriers with phonons, carriers and impurities. With the general rule established, the measurement of spin-based phenomena could give information on the carrier identity.

In our experiments, according to the spin-related optical transition rule, the excitons with an angular momentum of |+1⟩ and |−1⟩ in pure spin states can be selectively excited by left (*ϕ*^+^) and right (*ϕ*^−^) circular polarized pump pulses [19,21]. Using similar measurements for all samples, we present the transient reflection (TR) spectra for same circular (SC) and counter circular (CC) of the *n* = 1 sample as an example, shown in Fig. 1c and d. Additionally, a distinct difference in photoinduced absorption (PA) signals between the CC and SC spectra at 500 nm (PA1) and 534 nm (PA2) indicates the generation of different spin polarization. Figure 1e–g show the time-dependent spin population of the exclusively active excitons. The spin relaxation lifetime is determined by fitting the time dependence of difference signals, Δ*R*/*R*(Diff) = Δ*R*/*R*(SC) − Δ*R*/*R*(CC), using a single exponential fit to the time decay, demonstrating the spin lifetimes (${{\tau }_s}$) of 24.3, 8.4 and 8.4 ps for *n* = 1, 2, 3, respectively (Fig. 1h).

The observed layer-dependent *τ*_s_ here is quite different from that in the literature, where ${{\tau }_{\mathrm{s}}}$ in 2D perovskite were measured at room temperature, showing a gradual increase in ${{\tau }_s}$ with increasing *n* value [21], thereby diminishing the significant contribution from EY scattering mechanisms. On the other hand, pure DP relaxation through intrinsic SOC alone could also not fully explain the trend either, as the relaxation lifetime is correlated with exciton binding energy *E*_b_ with ${{\tau }_{\mathrm{s}}}$^−1^∼*a* + *bE*_b_^2^ [23]. The DP mechanism plays a role in spin relaxation by effectively changing the spin state through both the strength of the exchange interaction and the momentum scattering time. And the exchange interaction in excitons is influenced by effect of a magnetic field or an electric field, according to the exciton spin dynamics theory from Maialle-Silva-Sham [24]. In the quantum wells situation, the DP mechanism drives spin flipping through exchange interaction, and this exchange interaction is enhanced by the confinement of the quantum well, which affects the exciton spin relaxation time. Conventional 2D perovskites usually have a large binding energy and a small dielectric constant, indicating the dominance of the Coulomb exchange interaction in governing their spin relaxation. However, the spin relaxation behavior suggests that the *n* = 1 sample has an unusual spin protection mechanism, which could lead to the suppression of DP interaction and a long spin lifetime. By contrast, materials with a strong electron–phonon interaction (EPI) property, e.g. Cs_2_AgBiBr_6_ and CsPbBr_3_, are likely to be less sensitive to the Coulomb potential due to their dynamic lattice screening [11,13]. Therefore, our unusually long spin-polarized lifetime for *n* = 1 4AMP-based DJ perovskites might also originate from the dynamic lattice that could potentially lead to polarons which change the SOC ($\frac{1}{{{{\tau }_{{\mathrm{SOC}}}}}}$). At high carrier density, the scattering rate increases and spin lifetime decreases (Figs S8–S11). The decrease for *n* = 1 is much larger compared with *n* = 2 and 3, indicating a possibly stronger electron-phonon scattering ($\frac{1}{{{{\tau }_{{\mathrm{scattering}}}}}}$), which can be a prerequisite for polaron formation. Next, we will focus on the discussion of short-range EPI and polaron formation in 4AMP samples and provide evidence that the spin lifetime enhancement originates from the small polaron effect.

### Giant deformation potential induced small polaron effect

**Figure 2.**
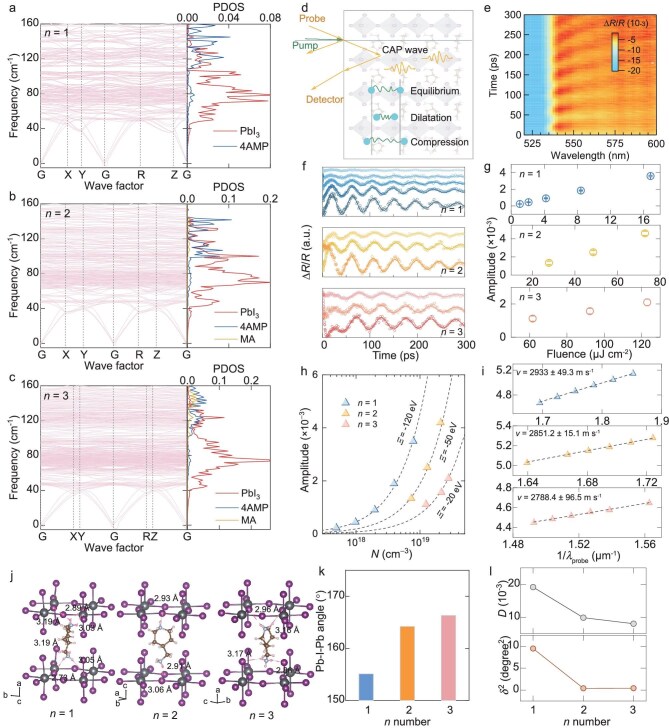
(a–c) Calculated phonon dispersions and the corresponding phonon density of states of *n* = 1, 2 and 3 sample. (d) Schematic of the generation of coherent acoustic phonon (CAP) wave and deformation potential model. (e) Pseudo color map of TR spectrum at a delay of 3 ps of *n* = 1 excited at 515 nm with a fluence of 8.42 μJ cm^−2^. (f) Pump fluence-dependent Δ*R*/*R* for CAP oscillation after subtracting contributions for these three samples. Fitting parameter of the CAP wave amplitude according to the strain function in the Supplementary Data. (g) The oscillation amplitude as a function of the pump fluence. (h) CAP amplitude functions of carrier concentration (the dashed lines represent Hooke's law with different deformation potentials). (i) Acoustic phonon wave velocity, which, according to $\frac{f}{{2\sqrt {{{n}^2} - {\mathrm{si}}{{{\mathrm{n}}}^2}\theta } }} = \frac{v}{{{{\lambda }_{{\mathrm{probe}}}}}}$, can be calculated by the fitted slope, and strain function in the Supplementary Data, respectively. Comparison of crystal distortion, including (j) different hydrogen bonding, (k) average axial and equatorial angles, (l) distortion index and bond angle variance parameter.

We first conducted density functional perturbation theory calculations to compute the phonon dispersion relations, phonon density of states, and projected (element-specific) phonon density of states (PDOS). As depicted in Fig. 2a–c, our analysis of the phonon dispersion relations within the first Brillouin zone revealed that samples with *n* = 1–3 exhibited low-lying longitudinal optical (LO) phonons in the frequency range of 45–160 cm^−1^, primarily attributed to vibrations associated with [PbI_6_]^4−^. Intermediate and high-energy phonon modes were mainly attributed to the 4AMP^2+^ and MA^+^ organic cations (as indicated by the PDOS data in Fig. S12). From the computed results, the optical phonon dispersion seems to be dominated by the [PbI_6_]^4−^; to further study the EPI, we turned our eye to the acoustic phonon, which can map the short-range EPI information by monitoring the time-resolved electronic structure and coherent acoustic phonon (CAP) dynamics [18].

Coherent phonon generation, detected from TR measurement, is depicted in Fig. 2d. Here, the above-bandgap laser pump excites electron and hole pairs, which impulsively heat the lattice through EPI and launch coherent phonon waves. Coherent phonon dynamics has recently been proposed as a method to characterize deformation potential (*Ξ*). And *Ξ is* a key metric for quantifying electron-phonon coupling in semiconductors according to Bardeen and Shockley's theory [25]. Figure 2e shows a transient Δ*R*/*R* pseudo color map for probe wavelengths between 520 and 600 nm and time delay within the 300 ps upon 515 nm laser pumping at a fluence of ∼8.42 μJ cm^−2^ in the *n* = 1 sample. Figure S13 presents the Fourier transform of the TR signals after removing the recombination process contribution, revealing the frequency of the Brillouin oscillation at ∼20 GHz in the *n* = 1 sample. The existence of a noticeable CAP wave originating from laser-induced lattice stress in *n* = 1 can be observed in the below-bandgap range. In a material, the total lattice stress $\sigma $ consists of two components [26]:


(2) \begin{eqnarray*}
\sigma = {{\sigma }_{{\mathrm{TE}}}} + {{\sigma }_{{\mathrm{DP}}}},
\end{eqnarray*}

where *σ*_TE_ and *σ*_DP_ are the thermoelastic stress and deformation potential stress, respectively. When resonant pumping is used with bandgap, the σ_TE_ term becomes very small and the *σ*_DP_ term stands out and it is defined by:


(3) \begin{eqnarray*}
{{\sigma }_{{\mathrm{DP}}}} &=& \mathop \sum \limits_k \delta {{n}_e}\left( k \right)\frac{{\partial {{E}_k}}}{{\partial \eta }} = N\frac{{\partial {{E}_{\mathrm{g}}}}}{{\partial \eta }} \\
&=& - NB\frac{{\partial {{E}_g}}}{{\partial P}} = - N\Xi ,
\end{eqnarray*}

where $\delta {{n}_e}( k )$ is the change of the electronic concentration at the level *k, η* is the strain, $\partial {{E}_k}/\partial \eta $ is the deformation potential parameter, *B* is the bulk modulus and *N* is the photoexcited carrier concentration.

The direct measurement of *Ξ* is quite difficult, so an iterative method was used to find *Ξ* based on the experimental CAP amplitude and carrier concentration (Supporting Information notes and Figs S14 and S15). Figure 2f shows the CAP dynamics at different pump fluences measured in the *n* = 1 sample, with similar measurements for *n* = 2 and 3 samples listed in Figs S16–S19. A strain function was employed to characterize the strength of lattice strain-induced deformation potential by fitting the oscillations of Δ*R*/*R* with a cosine function, giving the expression of the Δ*R*/*R* signal as [27]:


(4) \begin{eqnarray*}
\frac{{{\mathrm{\Delta }}R}}{R} = A{{{\mathrm{e}}}^{ - \frac{t}{{{{\tau }_{ph}}}}}}\cos \left( {2\pi ft + \theta } \right),
\end{eqnarray*}

where *A* is amplitude, *t* is the pump-probe delay, ${{\tau }_{ph}}$ is the CAP lifetime, *f* is frequency and $\theta $ is initial phase. When plotting the amplitude of these samples as functions of the power fluence, as shown in Fig. 2g, we found that the amplitudes of the CAP signals scale linearly with the power fluences, validating the calculation of *Ξ*. Figure 2h depicts the calculated *Ξ* as functions of carrier concentration in *n* = 1–3 samples to holistically investigate the intrinsic EPI properties. The EPI strength can be quantified by the *Ξ* value and fitting the experimental amplitude data demonstrated that the *n* = 1 sample in this work had a markedly large deformation potential of −123.46 ± 3.25 eV, while for *n* = 2 and 3 samples, *Ξ* is −51.31 ± 1.81 eV and −21.25 ± 3.47 eV, respectively. As a comparison, an RP 2D perovskite (DFPD)_2_PbI_4_ (DFPD is 4,4-difluoropiperidinium) was measured as having a deformation potential of −4.5 eV (Fig. S20), very close to the literature values of (PEA)_2_PbI_4_ (PEA is phenethylammonium) and 3D perovskite MAPbI_3_ [28]. It is worth pointing out that the obtained *Ξ* value is the highest one compared with those of reported typical perovskite systems, with deformation potentials of −28.4 eV for Cs_2_AgBiBr_6_, −3.93 eV for MAPbI_3_ and −4.3 eV for (PEA)_2_PbI_4_ [13,27,28].

Such a large deformation potential leads us to study the origins. Correlations between crystal distortion evolution and deformation potential have been proposed in the literature for SnSe [29] and Cs_2_AgBiBr_6_ [13]. Unlike conventional RP type and 3D perovskites, DJ-type perovskites have extensive hydrogen bonding from 4AMP and could contribute to the lattice distortion. The structural data of (4AMP) (MA)*_n_*_−1_Pb*_n_*I_3_*_n_*_+1_ samples are obtained from crystallographic information in the literature [30]. As shown in Fig. 2j, the *n* = 1 sample exhibits pronounced octahedral tilting due to the presence of shorter average hydrogen bonding. Here, average axial and equatorial Pb–I–Pb angles, distortion index (*D*) and bond angle variance parameter (*δ*) were selected as indicators for crystal symmetry analysis [31], as shown in Fig. 2k and l. The *n* = 1 sample displayed notably higher octahedral tilting compared with the others, indicating lower crystal symmetry. The *D* and *δ* data of 4AMP decrease from 19.2 × 10^−3^ to 8.0 × 10^−3^ and from 9.47 to 0.41, respectively, as the layer thickness increases from *n* = 1 to 3. The differences in structural features translate into a lower degree of structure symmetry (more distortion) in the *n* = 1 sample, leading to a giant deformation potential.

In the case of small polaron formation, a phase transition from a free state to a self-trapped exciton (STE) state occurs if the EPI strength is above a specific threshold value [15]. For example, Martin *et al*. [32] proposed an approximate coupling factor for characterization of STE with deformation potential measurement based on the theory of Toyozawa [33]. And the coupling constant *g* is given by:


(5) \begin{eqnarray*}
g = \frac{1}{6}\frac{{{{\Xi }^2}{{m}^*}}}{{{{\hbar }^2}{{C}_l}a}},
\end{eqnarray*}

where ${{m}^*}$ is the density state of effective mass, *C*_l_ is the longitudinal elastic constant and *a* is the lattice parameter. The sample that has larger *Ξ*/*C*_l_ factor exhibits higher STE probability. The longitudinal elastic constant can be calculated by *C*_l_ = *v*^2^*d*, where *v* describes the sound velocity through $f = \frac{{2v\sqrt {{{n}^2} - {\rm sin}^{2}\theta } }}{\lambda_{probe}}$ and *d* is the sample density [28,34]. Using the CAP frequency and refractive index at each probe wavelength, the fitting results suggested a *C*_l_ of 28.8, 29.8 and 29.6 GPa for *n* = 1, 2 and 3 sample, respectively, as shown in Fig. 2i (Figs S15, S17, S19). Due to the large deformation potential in the *n* = 1 sample, the obtained *Ξ*/*C*_l_ factor of 4.28 × 10^−10^ eV Pa^−1^ for the *n* = 1 sample is much higher than that of the *n* = 2 and 3 sample. It is evident that with such a large deformation potential, the EPI constant increases significantly, facilitating the formation of small polarons.

### Multi-angle evidence for small polarons

**Figure 3.**
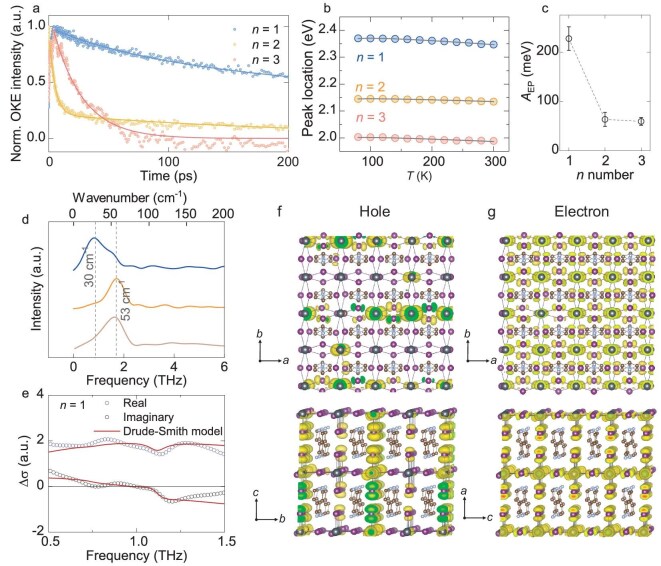
Small polaron properties characterization of *n* = 1–3 samples. (a) Time-resolved optical Kerr effect response measurement. (b) Photoluminescence peak locations as a function of the temperature. The solid lines represent the two Bose–Einstein oscillators model. (c) Exciton-phonon coupling strength (*A*_EP_) obtained the fitting parameters of the temperature-dependent peak location. (d) Fast Fourier transform (FFT) of the oscillation observed for *n* = 1–3 samples. (e) Complex THz photoinduced reflection spectra after excitation at 515 nm for *n* = 1 sample. The calculated charge distribution of (f) holes polaron and (g) electron polaron in 4 × 4 × 1 supercell of *n* = 1 samples.

Small polaron formation was further investigated with transient Optical Kerr Effects (TR-OKE) measurement. This technique utilizes the induced birefringence in the sample by a polarized optical field and detects the transient birefringence decay by controlling a time-delayed second optical field [7]. This process reveals the rotational motion characteristics of molecular ions in the sample (Fig. 3a and Fig. S21). Analysis of the TR-OKE data shows that in samples with *n* = 2 and *n* = 3, both instantaneous electronic and ultrafast responses were observed. However, in the *n* = 1 sample, in addition to these responses, long-term responses were also observed, covering a wide time window. This suggests that polaron motion plays a role in protecting charge carriers. Compared with the rapid decline of the OKE signal in the *n* = 2 and *n* = 3 samples, the OKE intensity in the *n* = 1 sample exhibited a flat decline curve and had a long-lived OKE lifetime of 652.6 ± 6.55 ps, indicating more lattice distortion. This can be explained by the photoinduced reorientation motion of dipolar molecular relaxation due to more prominent distortion in the *n* = 1 sample [7].

Because the formation of small polarons is driven by strong interaction between excitons and the lattice, we performed photoluminescence (PL) measurements under varying temperatures from 80 to 300 K (Fig. S22) to extract EPI strengths for these samples. Figure 3b displays the temperature dependence of the PL peak locations of our samples, with fits assuming two Bose–Einstein oscillators [35]. The bandgap of the samples decreases gradually with increasing temperature, showing typical heavily doped semiconductor behavior due to electron-phonon coupling. Figure 3c shows the electron-phonon coupling strength, indicating *n* = 1 has the largest coupling, correlating the carrier density-dependent spin relaxation discussed in the first section (Fig. S8).

The frequency shift of the phonons could serve as additional evidence for polaron formation. The phonon frequency could be extracted from coherent oscillation in the below bandgap region upon photoexcitation. Figure 3d and Figs S23–S25 show the extracted main frequency of *n* = 1–3 samples. For *n* = 2 and 3, the main peak is located around 1.5–1.6 THz (∼50 cm^−1^), corresponding to the I–Pb–I bending mode as suggested by Raman measurement [36]. However, for *n* = 1, the fast Fourier transform (FFT) frequency shifts down to 0.9 THz (30 cm^−1^). Other *n* = 1 RP phase (DFPD) or DJ phase (3-AMP) 2D perovskites also exhibit a 1.5–1.6 THz peak (Figs S26 and S27). Such a softening in the lattice has been attributed to small polaron formation in the B_12_C_3_ semiconductor [37]. Note that the measured 30 cm^−1^ value for *n* = 1 is a little different than the density functional theory (DFT) calculated value, which is in the ground state. This might also indicate that excited state charges are affecting the lattice degree of freedom for *n* = 1. Therefore, the difference in FFT frequency observed for *n* = 1–3 might be related to small polaron formation. Time-resolved THz spectroscopy (Fig. 3e) for *n* = 1, recorded at 10 ps with 515 nm excitation, provides further evidence. The photoinduced spectra can be modeled with the Drude–Smith model with phonon modification [38,39], which also could be related to polaron formation [40].

To visualize the size of the formed polaron, the corresponding features are investigated from first principles. As shown in Fig. 3f and g, the charge distribution of the highest valence state and the lowest conductive state in a locally distorted lattice (*n* = 1) corresponds to hole polarons and electron polarons, respectively. Here, the local distortions relate to the tiny expansion of four Pb–I bonds in a single Pb–I octahedron. These two types of charge carriers, holes and electrons, show significantly different features in a 4 × 4 × 1 supercell. Holes are found to localize in a small region within around 2-unit sizes of the perovskite cell (small polaron), while photo-generated electrons are found to show more delocalized behavior (large polaron). The difference in the localization picture makes it clear that the small polarons observed in optical measurements are mainly due to holes. Although calculations suggest hole polarons, based on our exciton binding energy, exciton polarons are more likely to be the main charge carriers. However, because the holes are trapped more easily, the exciton polarons might come from the hole polarons in the beginning. The origin of these differences can be traced back to the electronic band structures of the pristine lattice (Fig. S28), where the flat conduction band along the G-X direction in the Brillouin Zone and relatively large dispersion for the top valence band at the Fermi level can be seen. For the *n* = 2 sample, such polaron features vanish in our calculated charge distributions, coinciding with our experimental results (Fig. S28).

### Impact with temperature

**Figure 4.**
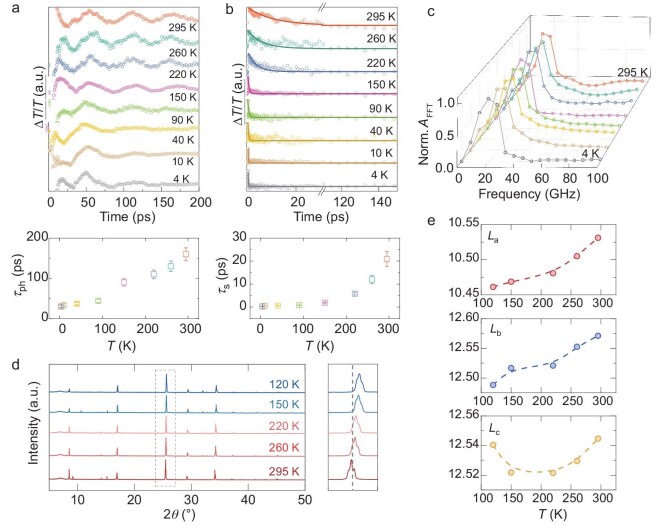
(a) CAP at a few typical temperatures ranging from 4 to 295 K and (bottom) temperature-dependent CAP lifetime obtained from strain function fitting. (b) Spin relaxation dynamics at different temperatures and (bottom) temperature dependence of their spin lifetime. (c) Temperature dependence of FFT amplitude. (d) The low-temperature powder XRD with a magnified pattern from 25° to 26° (2*θ*°) in the temperature range of 120–295 K. (e) Temperature-dependent lattice parameters obtained from refinement.

Temperature can be a good variable to tune and further study the effect of small polarons. We synthesized a *n* = 1 single crystal film for transient measurements in the temperature range of 4–295 K to extract the data of ${{\tau }_{ph}}$ (coherent acoustic wave lifetime) and ${{\tau }_s}$ (spin lifetime). The crystal film exhibited the same structure as the bulk single crystal, as indicated by XRD and UV-Vis spectroscopy (Figs S29, S30). Transient deformation signals and spin dynamics are depicted in Fig. 4a and b, respectively (see Figs S31 and S32 for raw data). Figure 4c displays the temperature dependence of the acoustic phonon frequency from the normalized Fourier transform amplitude (*A*_FFT_). Although the frequency of the phonon mode did not change with temperature, the lifetime of CAPs, as well as the spin lifetime (from 22 to 0.2 ps), exhibited a sharp decrease with the decrease in temperature (bottom of Fig. 4a). This interesting observation further shows the importance of lattice–charge interaction, as decreasing temperature will ‘freeze’ the phonons and thus decrease the chance of polaron formation. Also, the spin-lifetime change with temperature is matching the temperature dependence of the lattice strain. In Fig. 4d and e, we performed temperature-variable powder XRD patterns and a zoom on the (300)-reflection (from 120 to 295 K) to understand structural evolution. The results indicated a constant monoclinic structure within the measured temperature range (Fig. S33), suggesting weaker chemical bonding at room temperature compared with that at low temperature due to thermal expansion. Continuous lattice thermal expansion in *n* = 1 led to increased lattice softening or geometry distortion, which played a dominant role in increasing deformation potential [29]. Therefore, due to temperature-induced small polaron dynamics, the spin lifetime increased with increasing temperature, with the highest lifetime exceeding 20 ps at 295 K.

## DISCUSSION AND CONCLUSION

**Figure 5.**
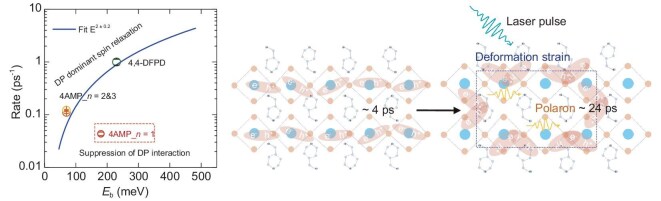
Comparison of spin-relaxation rate with theoretical curve and schematic diagram of small polaron effect.

We have established a way of combining deformation potential analysis, carrier spin relaxation, the optical Kerr effect and theoretical modeling to understand small polaron formation in perovskites. The large *Ξ* value of ∼−123 eV is expected to enhance the short-range charge phonon interaction of *n* = 1 (4AMP)PbI_4_, directly linking lattice deformation to charge carrier degrees of freedom. The discovered polaron has several interesting implications in future perovskite optoelectronics. The most interesting implication is found for perovskite-based spintronic devices. Polarons can benefit spin lifetime through tuning SOC and charge lattice interaction. The proposed deformation potential/longitudinal elastic constant (*Ξ*/*C*_l_) factor and spin relaxation/exciton binding energy can serve as a guideline for a new design. Figure 5 shows a comparison of spin relaxation rates due to exciton binding energy in the typical DP interaction model at room temperature. Generally, the spin relaxation rate follows exciton binding energy with: ${{\tau }_{\mathrm{s}}}$^−1^∼*a* + *bE*_b_^2^, where *E*_b_ is the exciton binding energy. The spin lifetime becomes shorter for materials with larger exciton binding energy, as the magnitude of Coulomb exchange interaction becomes relatively stronger. In the *n* = 1 sample with *E*_b_ = 180 meV, a corresponding ${{\tau }_s}$ ≈ 4 ps at room temperature is expected. However, in the *n* = 1 sample, with a large deformation potential/elastic constant (*Ξ*/*C*_l_) of 4.28 × 10^−10^ eV Pa^−1^, small polarons were formed. As a result, the Columbic exchange interaction between excitons could be screened by lattice and the distorted lattice could tune the SOC $\langle {{\mathrm{\Omega }}_k^2} \rangle $ to achieve a longer spin-polarized lifetime predicted by theory (Supporting Information Notes 4). For *n* = 2 and 3, because of the lack of polaron formation, the lifetime follows the theoretical line described in Equation (4). Such a picture is also depicted in Fig. 5.

In addition, the strong short-range electron–phonon interaction induced by polarons could potentially benefit perovskite-based hot carrier devices. Hot carrier relaxation can benefit from the hot phonon bottleneck effect, which depends on the phonon emission rate 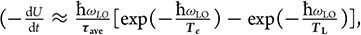 where ${{{{\tau }}}_{{{\rm ave}}}}$ is the average scattering time and ${{\omega }_{{\mathrm{LO}}}}$ is the phonon frequency) [41–43]. A strong electron–phonon interaction could lead to an elongated hot carrier lifetime [44,45]. By tuning the charge phonon coupling through deformation potential, one can expect a fine tuning of hot carrier lifetime. Because DJ phase 2D perovskites can have much more hydrogen bonding, which could induce more lattice distortion than RP phase 2D perovskites, there is a higher chance that hot carrier devices can be achieved in DJ perovskites. Lastly, attention also needs to be given to the transport properties of polarons. The hopping mechanism of polarons and band-like transport of semiconductors need to be considered when designing perovskite-based optoelectronic devices for future applications.

## MATERIALS AND METHODS

### Materials

PbO (99%, Alfa Aesar), hydroiodic acid (≥47%, Macklin), hypophosphorous acid (50 wt % in H_2_O, Aladdin), 4-(aminomethyl)piperidine (96%, Bidepharm) and methylamine hydrochloride (98%, Macklin) were purchased commercially and used without any further purification or modification. The detailed synthesis of the various compounds is presented in the supporting information.

### Characterization

Phase characterization of the single-crystal samples was performed by powder XRD (Cu K*α* radiation) using a PANalytical X'Pert Powder apparatus. The scanning range was 5–90 degrees, and the scan increment was 0.1 degree. The exciton binding energy of the samples was estimated by the Kubelka–Munk relationship *F*(*R*) = *K*/*S* = (1 − *R*)^2^/*R*, where *K* is the molar absorption coefficient, *S* is the scattering and *R* is the reflectivity determined by a diffuse reflection method from 200 to 1000 nm on the UH5700 UV-Vis spectrophotometer. BaSO_4_ was used as a non-absorbing reflectance reference. Temperature-dependent (80–300 K) PL spectra were acquired with an excitation wavelength of 410 nm on a FLS1000 Spectrometer-Edinburgh instrument.

### Transient spectroscopy measurements

Transient spectroscopy was measured with a pump probe set-up from a Ti-sapphire laser (Coherent Astrella) for both pump and probe pulses. A fraction of the fundamental 800-nm beams was used to generate the pump pulses with an optical parametric amplifier (TOPAS, Lightconversion), and another fraction of the beams was focused into a CaF_2_ crystal to generate the probe pulses (325–800 nm). The probe size is ∼180 μm × 125 μm, and the beam size of the 480 nm pump is ∼440 μm × 340 μm. Transient signals were collected by using a Timetech TA-100 spectroscopy system. The photoinduced spin-relaxation dynamics was performed using a circularly polarized pump and probe pulse (generated by passing the beam through a quarter waveplate (Thorlabs)). SC and CC polarized pump/probe measurements were collected. For low temperature measurement, the sample was placed in a closed-cycle cryostat under high vacuum (∼0.1 Pa) in the temperature range of 4–295 K. The pump power was set as 68.9 μJ cm^−2^. The detailed THz and OKE methods are presented in the supporting information.

### Calculation

In our calculations, we performed DFT by applying the Vienna Ab initio Simulation Package (VASP) code. The interactions between nuclei and the valence electrons are described by the projector augmented wave method [46]. Interactions between valence electrons are counted by the generalized gradient approximation [47] with the PBEsol formalism [48]. The basis set cut-off was 450 eV, and the *k*-space integration was done with a 4 × 4 × 4 *k*-mesh in the Monkhorst–Park scheme for formula unit cell and equivalent density for supercell was used. All the structures considered in this study were relaxed with a conjugate-gradient algorithm until the energy of the atoms was less than 3.0 × 10^−4^ eV/Å; the van der Waals interactions are taken into account in calculations using the DFT-D3 method [49]. The vibrational spectra and partial density of states are simulated through the phonopy code package [50] with interatomic forces calculated from VASP to evaluate its dynamical stability and the contribution of different components.

## Supplementary Material

nwae461_Supplemental_File

## DATA AVALABILITY

The datasets are available from the corresponding authors upon request.
