# Supplementary material for: Giant deformation potential induced small polaron effect in Dion–Jacobson two-dimensional lead halide perovskites
Source: Natl Sci Rev. 2024 Dec 16;12(5):nwae461. doi: 10.1093/nsr/nwae461 (PMC11970252; doi:10.1093/nsr/nwae461)
Supplement: nwae461_Supplemental_File [file nwae461_supplemental_file.pdf]

Supporting Information:
Giant deformation potential induced small polaron effect in Dion–Jacobson two-dimensional lead halide perovskites

Yuling Huang[1▽], Shaokuan Gong[1▽], Qianxia Chen[2,3▽], Congcong Chen[6], Zhangqiang Yang[7], Kang Wang[7], Jie Xue[8], Daozeng Wang[1], Haipeng Lu[8], Lingling Mao[6], Ye Yang[7], Jin-Zhu Zhao*[2,3,4,5], Xihan Chen*[1]

[1]Shenzhen Key Laboratory of Intelligent Robotics and Flexible Manufacturing Systems, SUSTech Energy Institute for Carbon Neutrality, Department of Mechanical and Energy Engineering, Southern University of Science and Technology, Shenzhen 518055, China.

[2]Guangdong Basic Research Center of Excellence for Structure and Fundamental Interactions of Matter, Guangdong Provincial Key Laboratory of Quantum Engineering and Quantum Materials, School of Physics, South China Normal University, Guangzhou 510006, P. R. China.

[3]Guangdong-Hong Kong Joint Laboratory of Quantum Matter, Frontier Research Institute for Physics, South China Normal University, Guangzhou 510006, P. R. China.

[4]Center for Computational Science and Engineering, Southern University of Science and Technology, Shenzhen 518055, P. R. China.

[5]National Laboratory of Solid State Microstructures, Nanjing University, Nanjing 210093, P. R. China.

[6]Department of Chemistry, Southern University of Science and Technology, Shenzhen 518055, China.

[7]Department of Chemistry, Xiamen University, Xiamen 361005, China.

[8]Department of Chemistry, The Hong Kong University of Science and Technology, Hong Kong 999077, China.

▽Y.H., S.G. and Q.C. contributed equally to this work.

Detailed Experimental Procedures

Materials:

PbO (99%, Alfa Aesar), hydroiodic acid (≥ 47%, Macklin), hypophosphorous acid (50 wt % in H2O, Aladdin), 4-(aminomethyl)piperidine (96%, Bidepharm), and methylamine hydrochloride (98%, Macklin), were purchased commercially and used without any further purification or modification. The detailed synthesis of the various compounds is presented in the supporting information.

Synthesis of $(4AMP)(MA)_{n-1}Pb_nI_{3n+1}$:

For the n = 1, PbO powder (669 mg, 3 mmol) was dissolved in a 6 mL of hydroiodic acid and 1.5 mL of hypophosphorous acid solution, heating with constant stirring for 5 min at 127°C until a clear yellow solution was obtained. 342 mg (3 mmol) of 4-(aminomethyl)piperidine (4AMP) was added to the previous solution under stirring and heating until complete dissolution. The solution was then allowed to cool to room temperature, resulting in the precipitation of orange plate-like crystals. For the n = 2, PbO powder (669 mg, 3 mmol) was dissolved in 6 mL of hydroiodic acid and 1.5 mL of hypophosphorous acid solution by heating with constant stirring for 5 min at 127°C until the solution turned clear. 57 mg (0.5 mmol) of 4AMP and 135 mg (2 mmol) of methylamine hydrochloride (MACl) were added directly to the previous solution under heating and stirring until complete dissolution. Red plate-like crystals precipitated when the solution was cooled to room temperature. For the higher layer-number (n = 3), it followed the same synthetic route except that the ratio was changed to 669 mg (3 mmol) of PbO, 38 mg (0.33 mmol) of 4AMP, and 202 mg (3 mmol) of MACl. Blackish dark red plate-like crystals were obtained during slow cooling to room temperature. For Single crystal film synthesis, the $(4AMP)PbI_4$ precursor solution was prepared by dissolving 60 mg sample in 20 μL dimethyl sulfoxide (DMSO) room temperature. Smooth and continuous single crystal film was fabricated by spin coating mixed precursor solution onto clean indium tin oxide (ITO) substrate at 2000rpm for 30 s. The film was dried immediately and annealed for 10 min at 373 K on a preheated hot plate.

Transient spectroscopy measurements.

Transient absorption and reflection spectroscopy was measured with a pump probe setup. where the fundamental beam (800 nm) is generated from a Ti-sapphire laser (Coherent Astrella) for both pump and probe pulses. A fraction of the 800-nm beams was used to generate the pump pulses with an optical parametric amplifier (TOPAS, Lightconversion), and another fraction of the beams was focused into a CaF_2 crystal to generate the probe pulses (325 nm-800 nm). The probe size is ~180×125 μm, and beam size of 480 nm pump is ~440×340 μm. Transient signals were collected by using Timetech, TA-100 transient spectroscopy system. The photoinduced spin-relaxation dynamics was performed using circularly polarized pump and probe pulse (generated by passing the beam through a quarter waveplate (Thorlabs) based on the transient reflection or absorption measurement. Same-circularly (SC) and counter-circularly (CC) polarized pump/probe measurements were collected for time

decay from 0-200 ps. For low temperature measurement, the sample was placed in a closed-cycle cryostat under high vacuum (≈10^{-6} Bar) for TAS measurements in the temperature range 4-295 K. The pump power was set as 68.9 μJ cm^{-2}.

Time-resolved Terahertz spectroscopy (tr-THz) setup was based on a Ti:Sapphire laser system (Coherent, Astrella). The THz pulse (0.5-2.0 THz) was generated by optical rectification via focusing the beam of 800 nm onto a ZnTe crystal. After that, it was refocused onto sample by parabolic mirror. Based on electro-optic sampling, the transmitted THz pulse was probed by a gating pulse of 800 nm at another ZnTe crystal. The pump beam of 515 nm from OPA (OPerA Solo, Coherent) was chopped to 500 Hz and overlapped with THz pulse in time and space on sample. The setup was closed in a box and purified by dry nitrogen to avoid disturbing from water absorption line. The power density was 0.2 mJ cm^{-2}.

The femtosecond TR-OKE measurements was powered by a femtosecond laser (YactoFiber, Hangzhou Yacto Technology Ltd), which generates 50 kHz 1030 nm pulse train with temporal pulse width of ~284 fs and with pulse energy of 100 μJ. A branch of the fundamental beam was split into two branches for pump and probe pulse (9 : 1), respectively. The optical pump pulse (515 nm) was generated via the second harmonic generation of the 1030 nm pulse in the barium borate (BBO) nonlinear crystal, and modulated by a mechanical chopper at 500 Hz, the residual 1030 nm beam was blocked by a 750 nm short-pass filter. The pump and probe were focused on the sample with polarizations set to 90° with respect to each other via Glan-Tylor polarizer, whereas a delay line was used to control the time delay between the pulses. The probe pulse transmitted through the sample was recollimated and split into two beams with vertical and horizontal polarizations by a Wollaston prism. The Kerr signal was detected by a balanced photodiodes (Newport, 2007) with 850 nm long-pass filter to eliminate any scattered light from the pump beam and recorded by a high-speed sampling DAQ device (National Instrument, PCIe-6374) synchronized with the modulation of the pump beam intensity. The spot size and pulse energy of the pump pulse was about 0.95 mm^2 and 30 nJ (1.5 mW).

Supporting Information Notes

Note 1: Binding energy calculation

The exciton binding energy (E_b) was computed based on Elliott formula for our 2D perovskite samples (1,2):

$$\alpha(h\nu) = \frac{A}{\zeta}\frac{\Gamma(E_b)}{\frac{\Gamma(E_b)^2}{4} + \left[\frac{h\nu - E_b}{\zeta}\right]^2} + \frac{B}{1 + \theta(h\nu - E_g)exp\left\{-2\pi\left[\frac{h\nu - E_g}{Ry}\right]^{-1/2}\right\}}\left\{\frac{1}{2} + \frac{1}{\pi}arctan\left[\frac{2(h\nu - E_g)}{\Gamma(E_g)}\right]\right\}$$

and

$$E_b = E_g - Ry$$

where A and B are the relative amplitudes of the exciton and free carrier absorptions, Ry is the Rydberg constant, and ζ and Γ are an energy-dependent and a density-dependent broadening parameter, respectively.

Then, we obtain the following expression to fit linear absorption spectra to extract exciton binding energy of synthesized samples:

$$\Gamma(E_x) = \frac{2(\Gamma_0)}{exp[-3(h\nu - E_x)/k_B T] + 1}$$

Note 2: Pump fluence and carrier concentration calculation

The pump fluence (F) and the carrier concentration (N) can be calculated input total energy of pump pulse (E_{pump}) based on the equation below:

$$\mathrm{F} = \frac{E_{pump}}{S}$$

$$\mathrm{N} = \frac{E_{pump}}{E_{photo} \times S \times D}$$

where E_{photo} is the energy of each photo; S is the pump beam size; and D (D = 1/α) is the penetration depth determined by the absorption coefficient of materials.

Note 3: Spin relaxation time calculation

By fitting this curve using a single-exponential equation, the spin relaxation time ($\tau_{1/2}$) can be obtained. This constitutes the basic approach of using circularly polarized transient dynamics to measure the spin relaxation time of 2D perovskites (2-4):

$$y = y_0 + A \exp\left(\frac{-(x - x_0)}{\tau_{1/2}}\right)$$

Note 4: D'yakonov-Perel' spin relaxation mechanism

The D'yakonov-Perel (DP) scattering occurs when spin procession is related to the scattering events by an effective Zeeman field, where the spin polarization is perturbed by the spin-orbit interaction in systems lacking inversion symmetry. In materials without inversion symmetry, the momentum state for spin-up and -down electrons is splitting, such as GaAs and ZnSe semiconductors. For such materials, the spin relaxation rate can be approximated to the following formula (5,6):

$$\tau_{DP}^{-1} = \frac{2}{3}\frac{\int_0^\infty d\varepsilon\, \rho(\varepsilon)\overline{\Omega^2}\tau_p(\varepsilon)[F_+(\varepsilon) - F_-(\varepsilon)]}{\int_0^\infty d\varepsilon\, \rho(\varepsilon)[F_+(\varepsilon) - F_-(\varepsilon)]}$$

where $\rho(\varepsilon)$ is the density of state, Ω is the magnitude of spin-orbit splitting, and $F_{+/-}(\varepsilon)$ is the Fermi distribution function for electrons with spin up and down momentum.

In the case of exciton spin dynamics in quantum wells, the DP mechanism is dominated by exciton binding energy:

$$\tau_{DP}^{-1} \approx \langle \Omega_k^2 \rangle \tau_p$$

Note 5: Elliott-Yafet spin relaxation mechanism

The Elliott-Yafet (EY) scattering occurs when phonon or ionized impurity wave moves through the lattice. It causes a Bloch electron in the crystal and lead to a perturbation of the energy bands and change in spin direction. The mathematical description for EY mechanism was based on the Eliashberg function $\alpha_S^2 F(\Omega)$, which can written as (5,6):

$$\tau_{EY}^{-1} = 8\pi T \int_0^\infty d\Omega \alpha_S^2 F(\Omega) \frac{\partial N(\Omega)}{\partial T}$$

where $N(\Omega) = \frac{1}{exp(\hbar\Omega/k_B T)-1}$ is the phonon distribution function.

With a simple Fermi-surface averaging assumption, the spin relaxation rate in the case of spin-flip scattering can be expressed as follows:

$$\tau_{EY}^{-1} \approx \langle b^2 \rangle / \tau_p$$

where $\langle b^2 \rangle$ is the spin-flip matrix element and τ_p is the momentum relaxation time.

Note 6: Deformation potential calculation

The lattice stress after photoexcitation of a material is evaluated by the amplitude of coherent acoustic phonon wave according to Hooke's law: $kA = \sigma_{\mathrm{TE}} + \sigma_{\mathrm{DP}}$, where k is simplified ratio parameter, A is the amplitude of coherent acoustic phonon, and σ_{TE} and σ_{DP} are the thermoelastic and deformation potential contributions to the total lattice stress, respectively. The deformation potential stress was calculated from the density of photoexcited carriers (N), deformation potential (Ξ) according to the relationship $\sigma_{\mathrm{DP}} = N\Xi$. In the case of our experiment, the σ_{TE} is relatively weak when a pump pulse with photon energy near band gap, thus we can neglect the thermoelastic contribution to the generation of total lattice stress (7-9). For simplicity, we can neglect the contribution from thermoelastic part under near-resonant pump ($h\nu - E_{\mathrm{g}} \approx 0$) measurement. The stress is directly proportional to the amplitude of the generated coherent acoustic phonons: $kA = \sigma_{\mathrm{DP}}$.

In a material, the total lattice stress (σ) consists of two components:

$$\sigma = \sigma_{TE} + \sigma_{DP}$$

where σ_{TE} and σ_{DP} are thermoelastic stress and deformation potential stress, respectively.

The deformation potential mechanism is the mechanism which relates the modification in energy of the electronic distribution to the strain in the solid. This is often written as:

$$\frac{\delta V}{V} = \frac{\delta U}{\Xi}$$

where Ξ is the deformation potential coefficient, U the electronic energy, V is the volume, and $\delta V/V$ is the strain caused by acoustic phonons. This equation underlines the direct link between the variation of the lattice strain and the electronic energy change.

According to Sommerfeld model, at thermodynamic equilibrium condition, the electronic pressure is defiend as:

$$P = \frac{2}{3}\frac{E}{\mathrm{V}}$$

where E is the total kinetic energy of free electrons.

When a material with a band gap (E_g) is photoexcited with a photon energy equal to the direct band gap, the deformation potential induced stress becomes can be calculated by:

$$\sigma_{DP} = \sum_k \delta n_e(k) \frac{\partial E_k}{\partial \eta} = N \frac{\partial E_g}{\partial \eta} = -NB \frac{\partial E_g}{\partial P} = -N\Xi$$

where $\delta n_e(k)$ is the change of the electronic concentration at the level k, η is the strain, $\partial E_k/\partial \eta$ is the deformation potential parameter, B is the bulk modulus, and N is the photoexcited carriers concentration.

To confirm our method and verify the reliability of our method to obtain the deformation potential, we applied similar approach to a material with well-known properties,

$(PEA)_2PbI_4$, and performed similar measurements. We can estimate the deformation potential of –4.8±0.4 eV for $(PEA)_2PbI_4$, which is in agreement with values reported in the literature of –4.3±0.2 eV. We have also done a measurement on $(DFPD)_2PbI_4$ (similar to the structure of $(PEA)_2PbI_4$)). And we found deformation potential of these materials very close to the literature values. We can now estimate the reflectivity and amplitude change induced by the stress, and furthermore, since we know σ_{DP}, we can then calculate Ξ.

Note 7: Calculations of electron phonon interaction strength

The theory of multi-Bose-Einstein oscillators model gives the expression of E_g as (10-12):

$$E_g(T) = E_0 + \sum_i A_i[2n_{BE}(E_i/k_BT) + 1]$$

$$n_{BE}(E_i/k_BT) = 1/[(E_1/k_BT) - 1]$$

where E_0 is the zero-temperature band gap, A_i is the weight which describes the degree of the energy decrease with increasing temperature, and n_{BE} is the Bose-Einstein factor and E_i is the corresponding fitted energy.

To examine the electron phonon interaction strength of the samples in this work, we assume that temperature dependence of E_g can be treated as the two Bose-Einstein oscillators. The E_g can be expressed as:

$$E_g(T) = E_0 + A_{TE}T + A_{EP}\left[\frac{2}{e^{\left(\frac{E_{ph}}{k_BT}\right)} - 1} + 1\right]$$

where A_{TE} and A_{EP} are the strengths of thermal expansion and electron phonon interaction, E_{ph} is longitudinal-optical phonon energy.

Meanwhile, E_{ph} can be calculated by:

$$E_{ph} = \hbar\omega_{LO}$$

where $\hbar$ is reduced Planck constant and ω_{LO} is longitudinal-optical phonon frequency.

Thus, E_{ph} data can be calculated by coherent phonon signal measurements. For both $n = 2$ and $n = 3$ samples, they possess similar E_{ph} ~6.6 meV, while E_{ph} of n = 1 sample is ~4 meV.

Fig. S1.

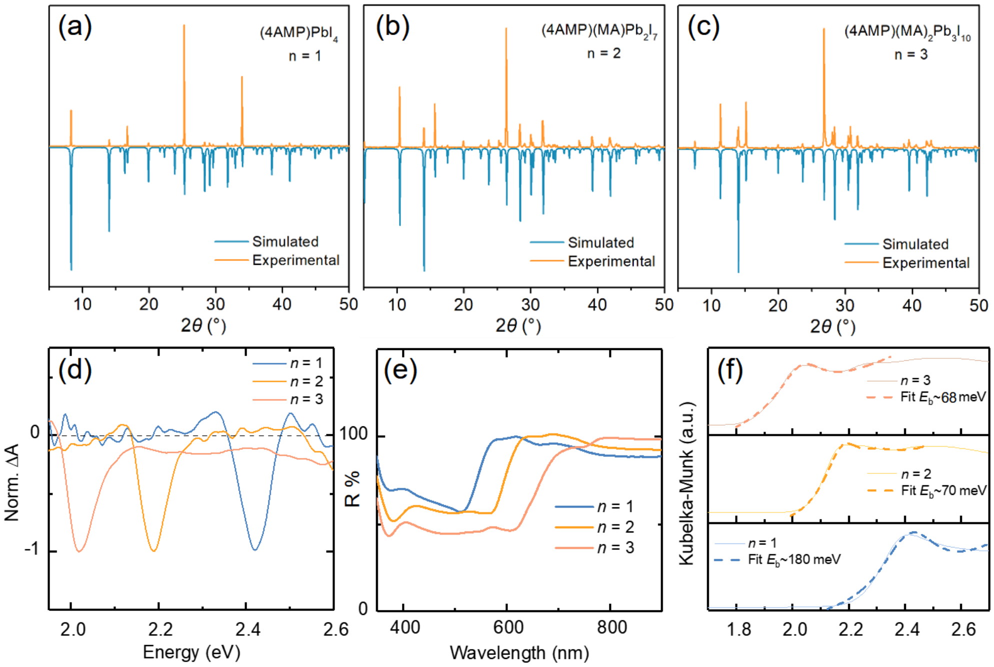

Fig. S1. (a), (b) and (c) Simulated and experimental PXRD for the $(4AMP)(MA)_{n-1}Pb_nI_{3n+1}$ (n = 1, 2 and 3). (d) Normalized transient spectra. The spectra are the Kramer-Kronig transformation of transient reflection spectra. (e) Optical reflection spectra. (f) Elliot formula to extract exciton binding energy of this serious, obtained from diffuse reflectance spectroscopy.

Fig. S2

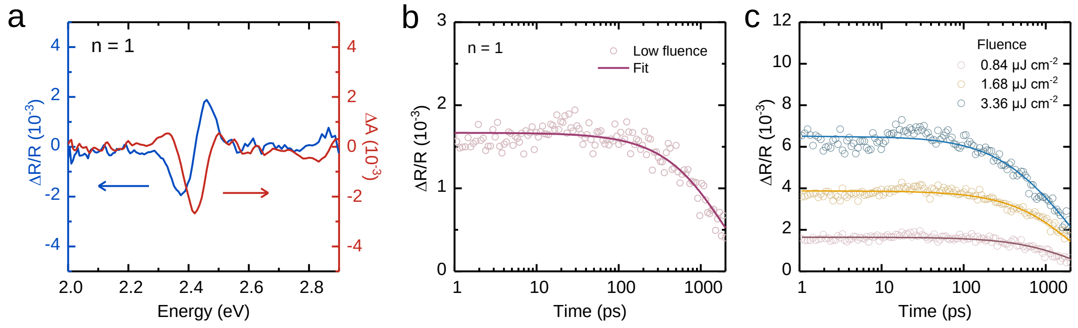

Fig. S2. TR spectra and recombination dynamics with pump energy of 2.58 eV in $(4AMP)PbI_4$ sample. (a) TR spectrum and its Kramers-Kronig transform spectra. (b) The kinetics at low pump fluence. The solid line show the single exponential decay fitting conducted for mono-molecular recombination coefficient extraction. The carrier lifetime of n = 1 sample is 1851.8±78.21 ps. (c) The recombination dynamics for different pump fluence. Solid lines represent fits using ABC model.

Fig. S3

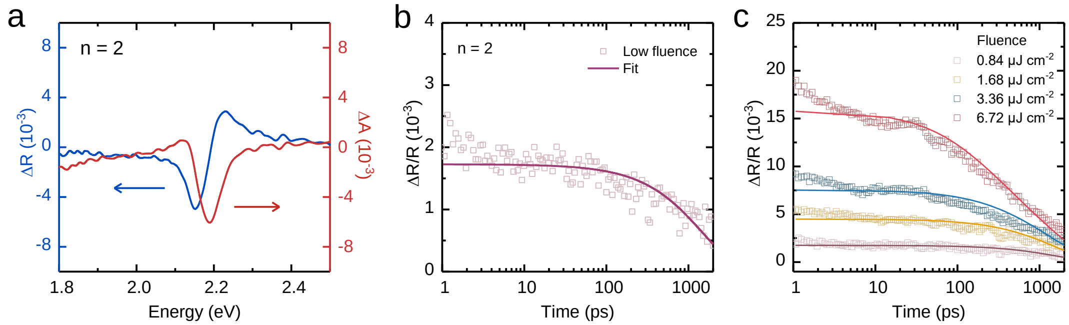

Fig. S3. TR spectra and recombination dynamics with pump energy of 2.58 eV in $(4AMP)(MA)Pb_2I_7$ sample. (a) TR spectrum and its Kramers-Kronig transform spectra. (b) The kinetics at low pump fluence. The carrier lifetime of n = 2 sample is 1164.5±80.25 ps. (c) The recombination dynamics for different pump fluence.

Fig. S4

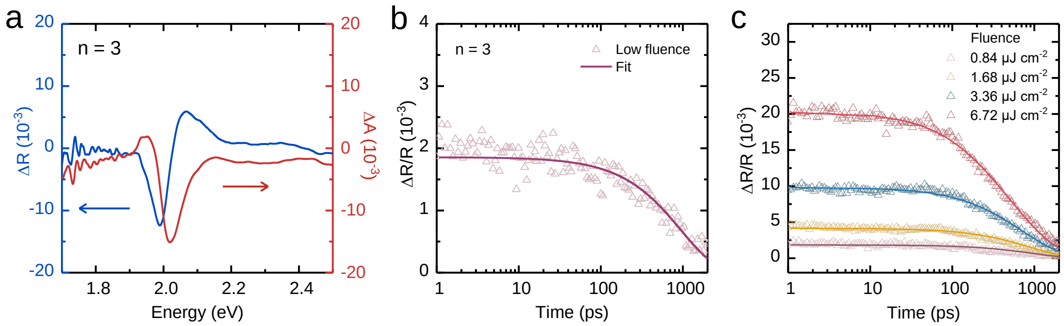

Fig. S4. TR spectra and recombination dynamics with pump energy of 2.58 eV in $(4AMP)(MA)_2Pb_3I_{10}$ sample. (a) TR spectrum and its Kramers-Kronig transform spectra. (b) The kinetics at low pump fluence. The carrier lifetime of n = 1 sample is 1057.1±49.44 ps. (c) The recombination dynamics for different pump fluence.

Fig. S5

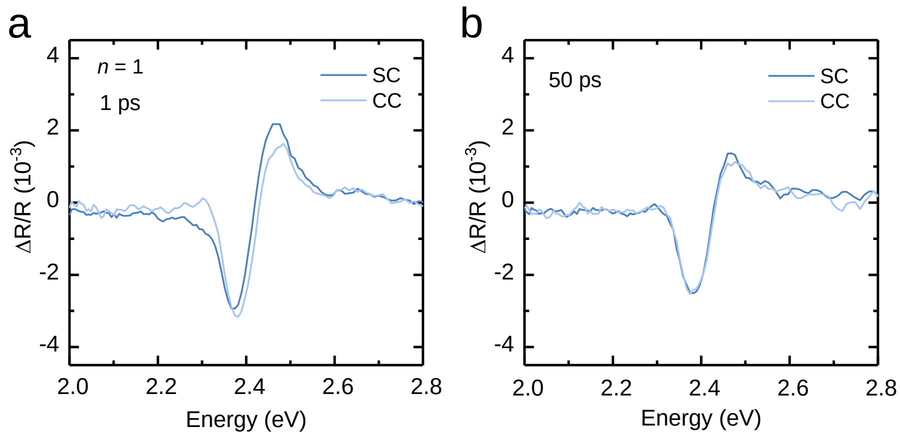

Fig. S5. Spectral evolution for SC and CC pump-probe at different 1 ps and 50 ps time delay for $(4AMP)PbI_4$ sample.

Fig. S6

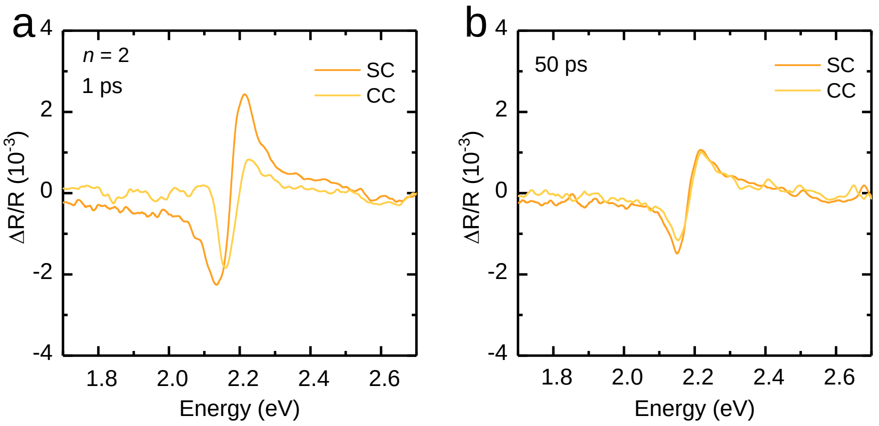

Fig. S6. Spectral evolution for SC and CC pump-probe at different 1 ps and 50 ps time delay for $(4AMP)(MA)Pb_2I_7$ sample.

Fig. S7

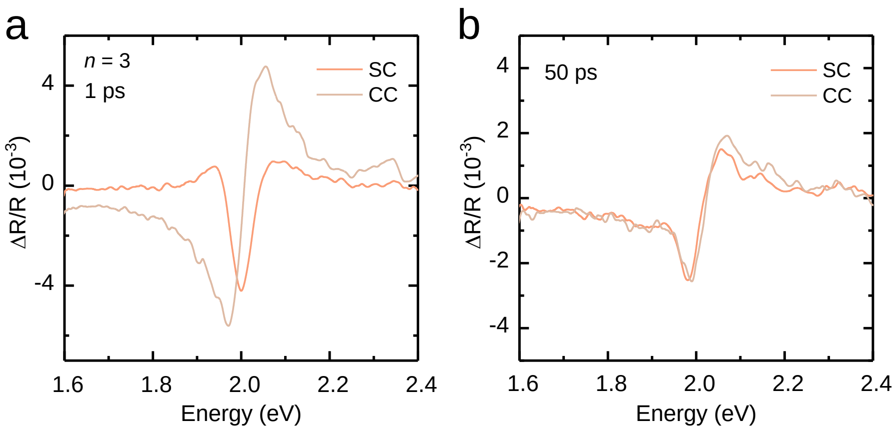

Fig. S7. Spectral evolution for SC and CC pump-probe at different 1 ps and 50 ps time delay for $(4AMP)(MA)_2Pb_3I_{10}$ sample.

Fig. S8

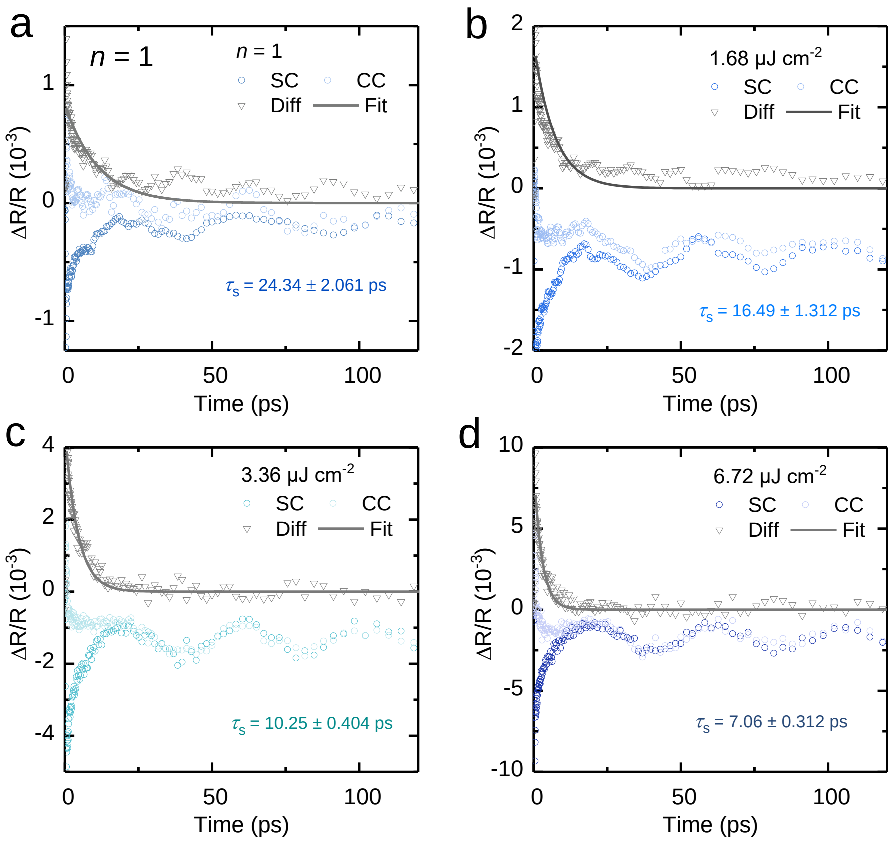

Fig. S8. SC and CC pump-probe induced spin dynamics of $(4AMP)PbI_4$ crystal as a function time delay, obtained at (a) 0.84, (b) 1.68, (c) 3.36 and (d) 6.72 μJ cm^{-2}. The solid grey curves are fits to the data with single exponential function.

Fig. S9

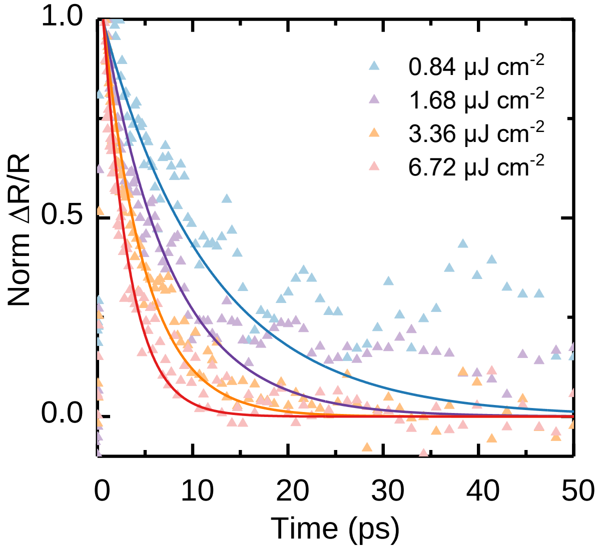

Fig. S9. Normalized spin relaxation kinetics at different exciton fluence shown in Figure S8 for $(4AMP)PbI_4$ sample.

Fig. S10

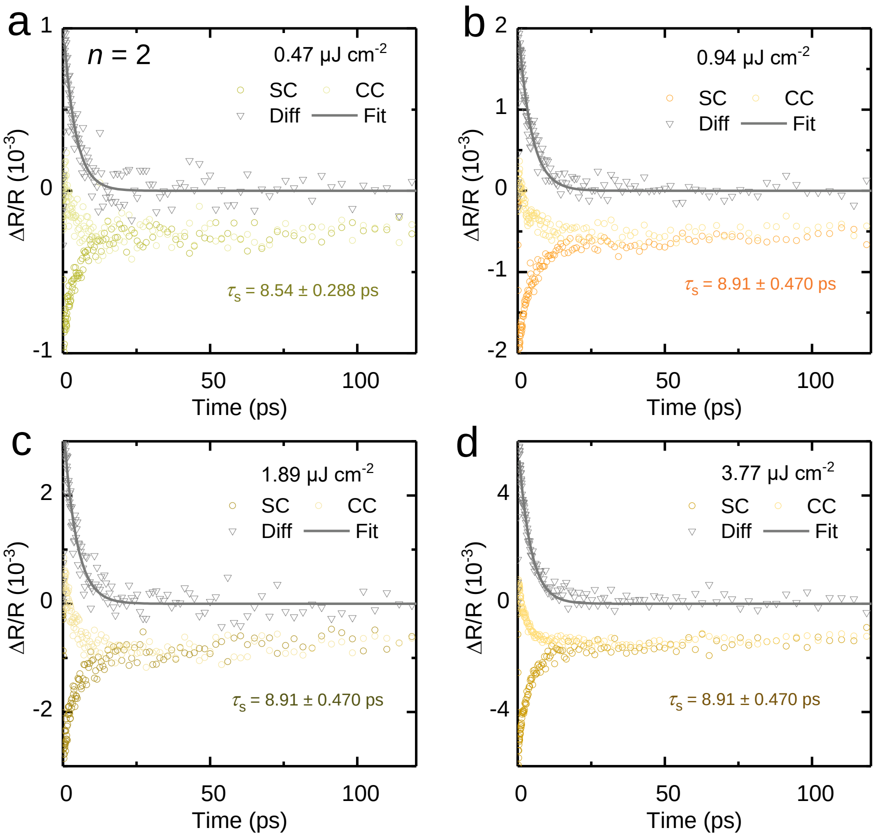

Fig. S10. SC and CC pump-probe induced spin dynamics of (4AMP)(MA)Pb_2I_7 crystal as a function time delay, obtained at (a) 0.47, (b) 0.94, (c) 1.89 and (d) 3.77 μJ cm^{-2}. The solid grey curves are fits to the data with single exponential function.

Fig. S11

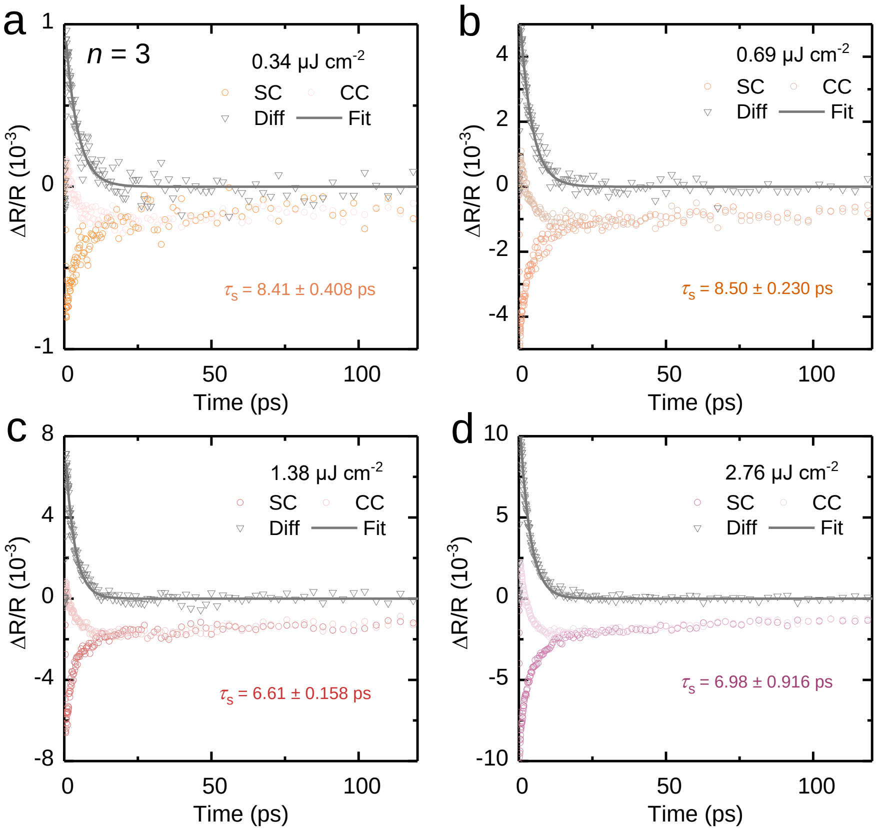

Fig. S11. SC and CC pump-probe induced spin dynamics of $(4AMP)(MA)_2Pb_3I_{10}$ crystal as a function time delay, obtained at (a) 0.77, (b) 1.55, (c) 3.10 and (d) 6.21 µJ cm^{-2}. The solid grey curves are fits to the data with single exponential function.

Fig. S12

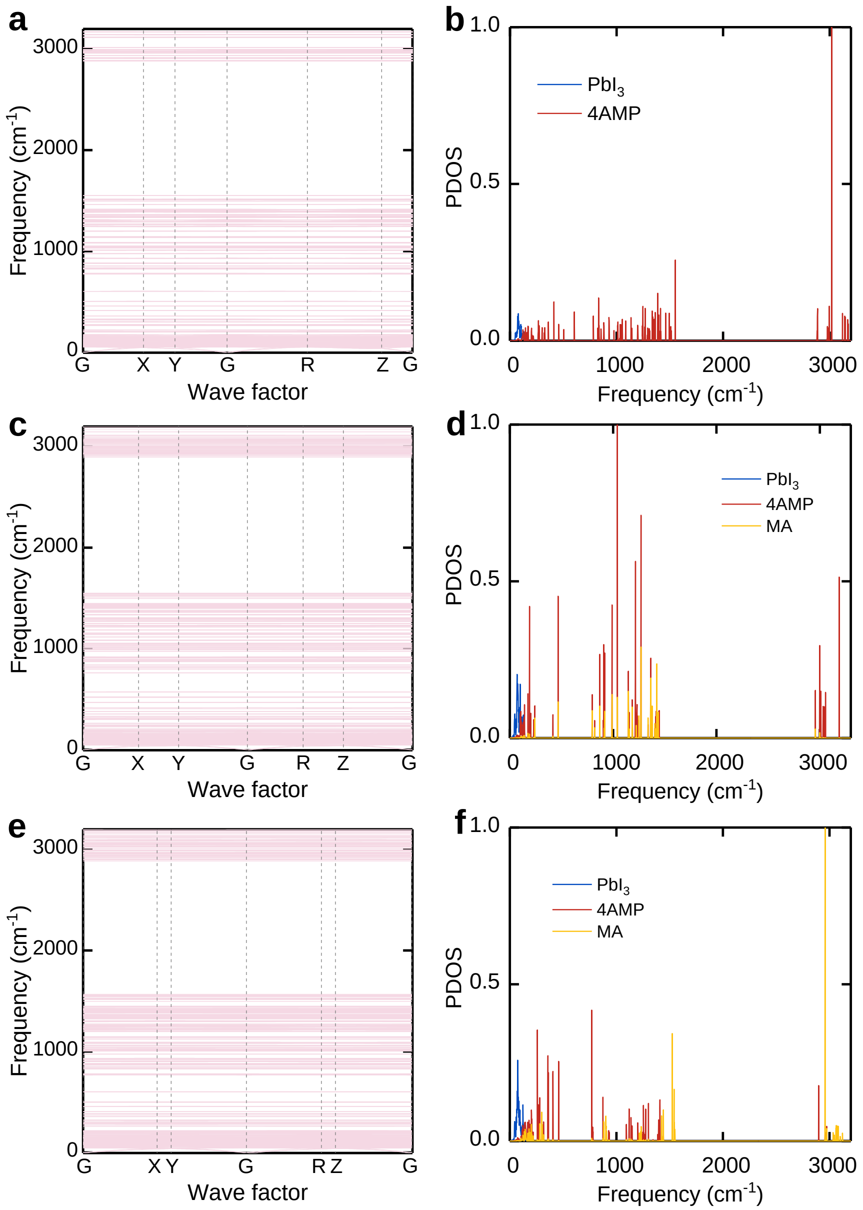

Fig. S12. Phonon dispersion curves for (a) $n = 1$, (c) $n = 2$, and (e) $n = 3$. Projected phonon density of states for (b) $n = 1$, (d) $n = 2$, and (f) $n = 3$.

Fig. S13

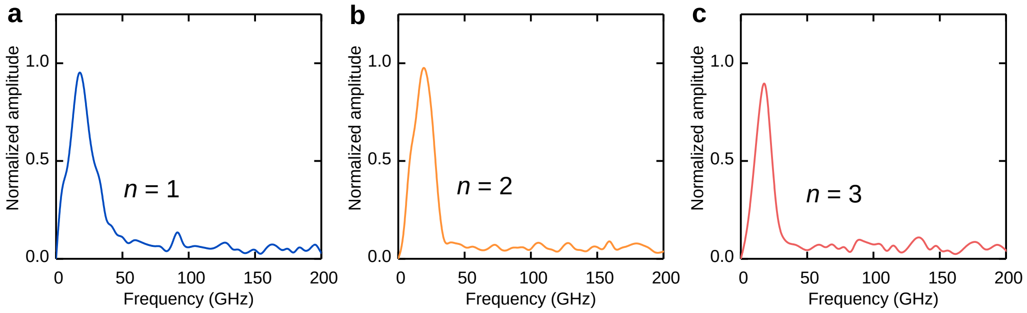

Fig. S13. Normalized Fourier transform amplitude of the coherent acoustic phonon oscillations for the $(4AMP)(MA)_{n-1}Pb_nI_{3n+1}$ (n = 1, 2 and 3).

Fig. S14

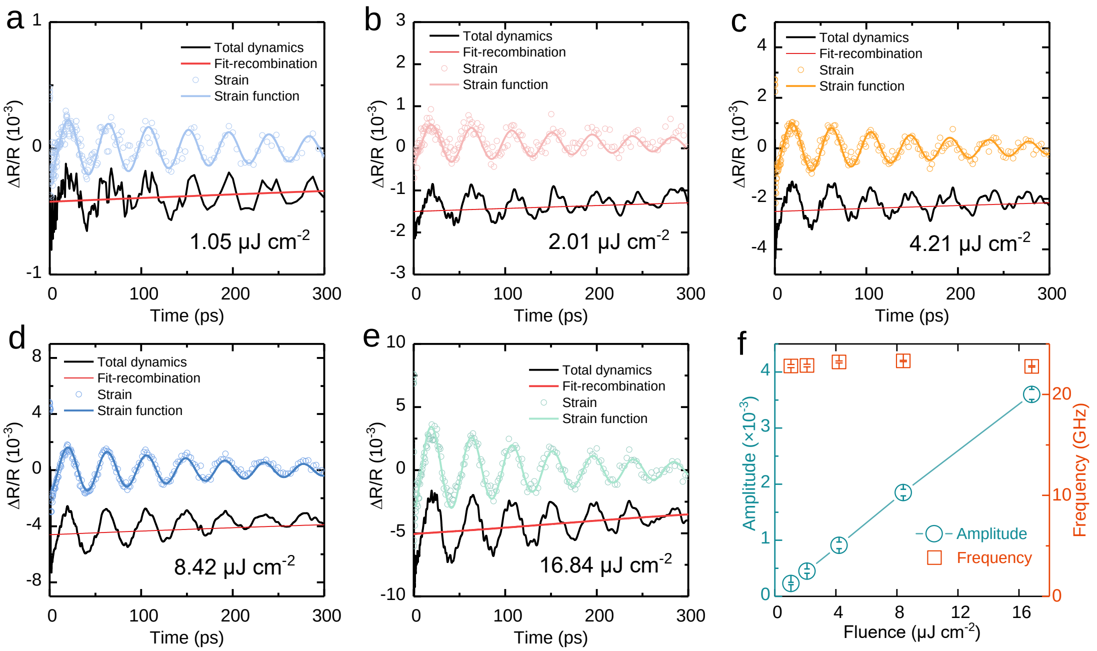

Fig. S14. Coherent acoustic phonon propagation in $(4AMP)PbI_4$ sample. TR kinetics signals at pump wavelength of 515 nm and a constant probe wavelength of 540 nm with a photo induced carrier concentration of (a) 4.89×10^{17}, (b) 9.77×10^{17}, (c) 1.95×10^{18}, (d) 3.91×10^{18} and (d) 7.82×10^{18} cm^{-3}. The change of the changing in exciton density results in the difference in amplitude.

Fig. S15

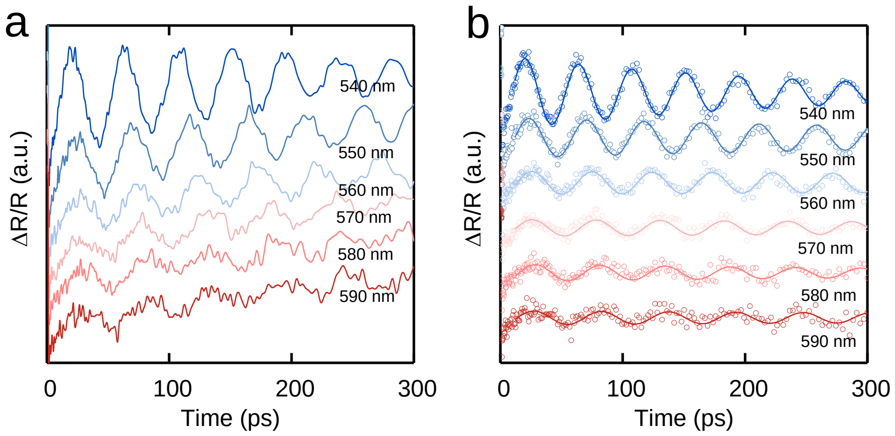

Fig. S15. CAP propagation velocity calculation in (4AMP)PbI_4 sample. (a) The transient reflectivity signals ($\Delta R/R$) probe at 540, 550, 560, 570, 580, and 590 nm, with a 515 pump at the fluence of 13.44 μJ cm^{-2}. (b) Oscillation parts of $\Delta R/R$ signals in (a).

Fig. S16

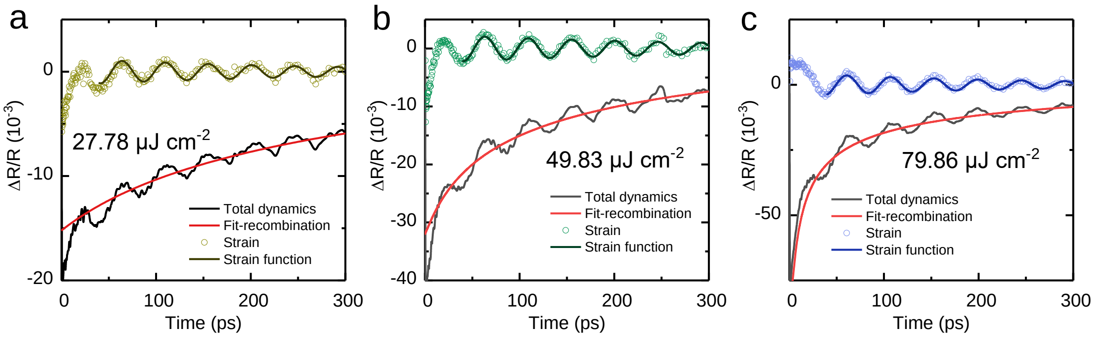

Fig. S16. Coherent acoustic phonon propagation in(4AMP)(MA)Pb_2I_7 sample. TR kinetics signals at pump wavelength of 560 nm and a constant probe wavelength of 590 nm with a photo induced carrier concentration of (a) 7.23×10^{18}, (b) 1.29×10^{19}, and (c) 2.08×10^{19} cm^{-3}.

Fig. S17

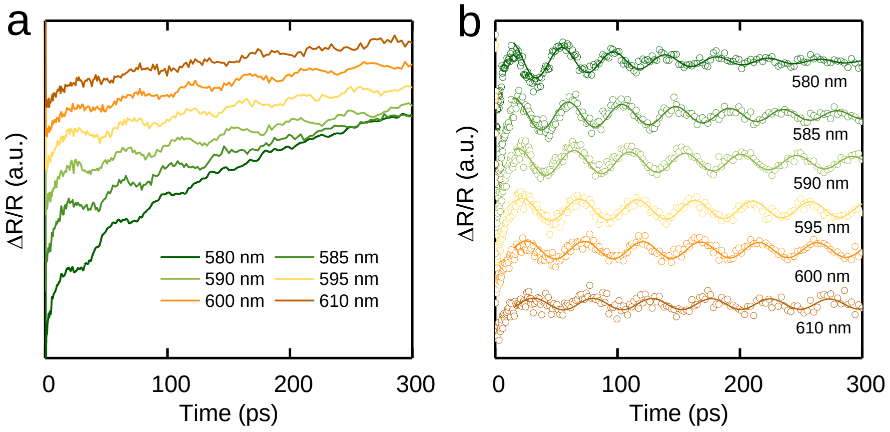

Fig. S17. CAP propagation velocity calculation in $(4AMP)(MA)Pb_2I_7$ sample. (a) The transient reflectivity signals ($\Delta R/R$) probe at 580, 585, 590, 595, 600, and 610 nm, with a 560 pump at the fluence of 27.78 μJ cm^{-2}. (b) Oscillation parts of $\Delta R/R$ signals in (a).

Fig. S18

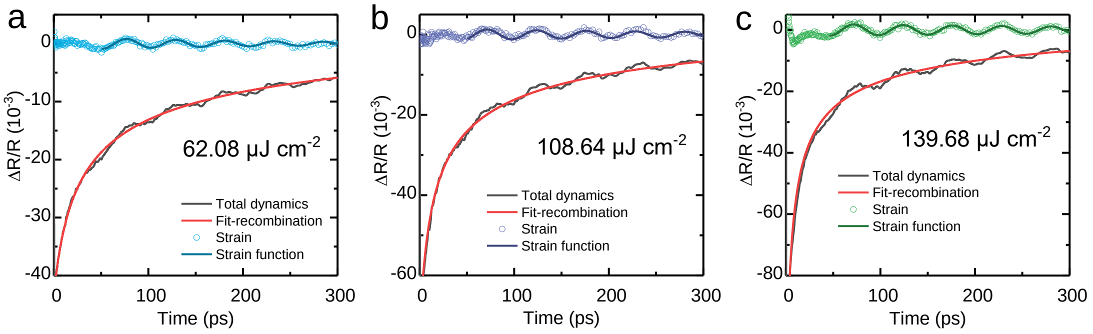

Fig. S18. Coherent acoustic phonon propagation in $(4AMP)(MA)_2Pb_3I_{10}$ sample. TR kinetics signals at pump wavelength of 630 nm and a constant probe wavelength of 650 nm with a photo induced carrier concentration of (a) 1.23×10^{19}, (b) 2.14×10^{19}, and (c) 2.76×10^{19} cm^{-3}.

Fig. S19

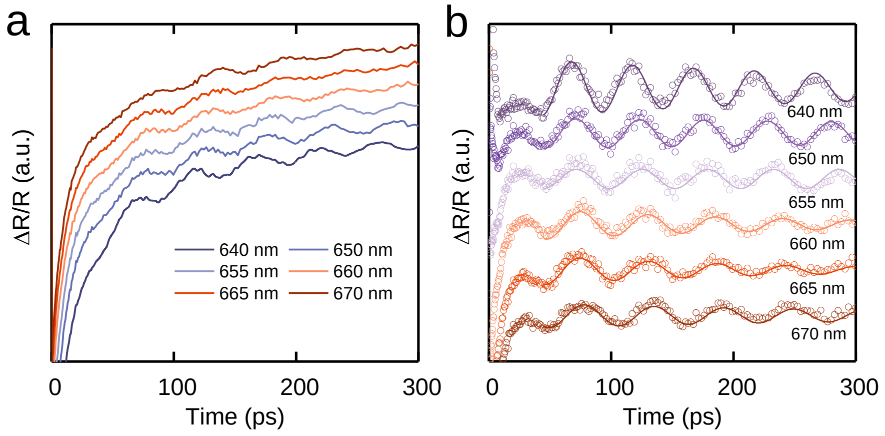

Fig. S19. CAP propagation velocity calculation in $(4AMP)(MA)_2Pb_3I_{10}$ sample. (a) The transient reflectivity signals ($\Delta R/R$) probe at 640, 650, 655, 660, 665, and 670 nm, with a 630 pump at the fluence of 62.08 μJ cm^{-2}. (b) Oscillation parts of $\Delta R/R$ signals in (a).

Fig. S20

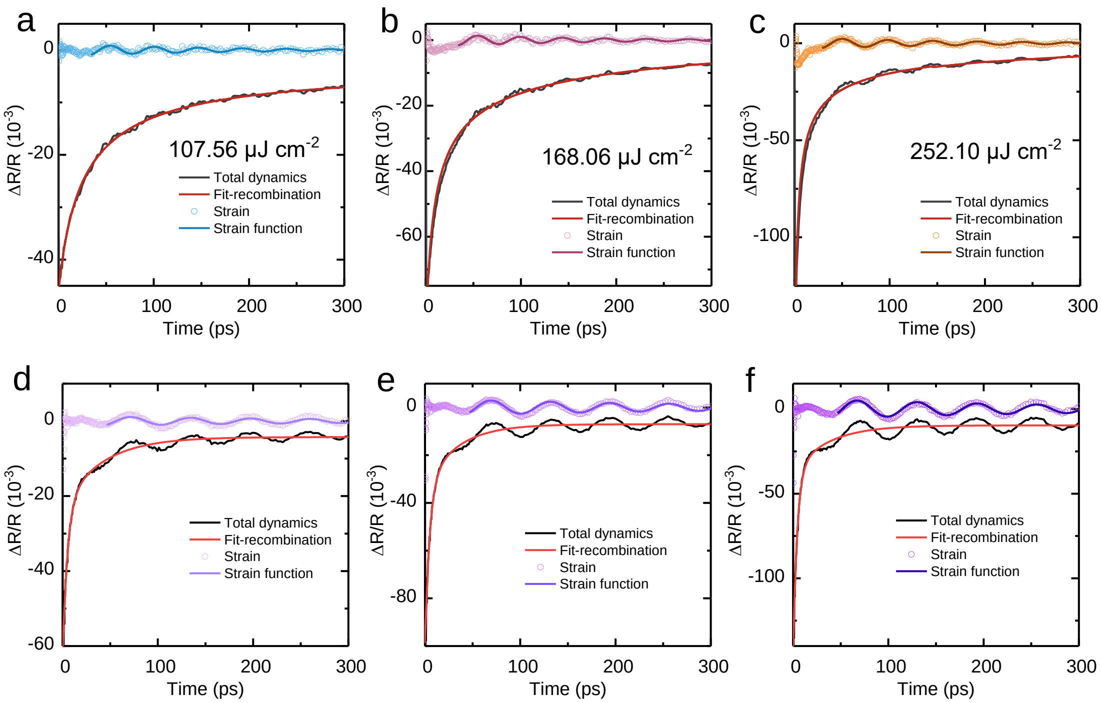

Fig. S20. Coherent acoustic phonon propagation in Ruddlesden-Popper phase (4,4-DFPD)$_2$PbI$_4$ (4,4-DFPD is 4,4-difluoropiperidinium) and (PEA)$_2$PbI$_4$. TR kinetics signals at pump wavelength of 510 nm with a photo induced carrier concentration of (a) 6.91×10^{19}, (b) 1.08×10^{20}, (c) 1.62×10^{20} cm^{-3}, (e) 7.49×10^{19} cm^{-3}, (f) 1.64×10^{20} cm^{-3} and (g) 2.85×10^{20} cm^{-3}.

Fig. S21

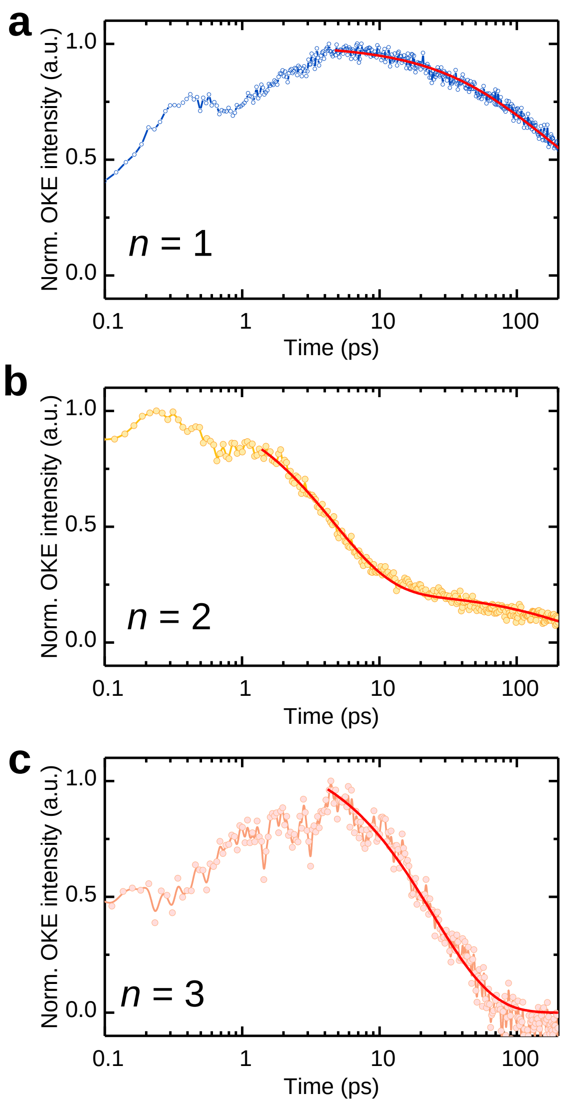

Fig. S21. Time-resolved optical Kerr effect (OKE) dynamics for n = 1-3 samples. The solid red curves are double-exponential fitting curve corresponding to the depolarization due to the polaron motion, which gives OKE response time constant of 652.6 ± 6.55 ps, 223.6 ± 10.70 ps and 25.36 ± 1.41 ps for n = 1, n = 2 and n = 3, respectively.

Fig. S22

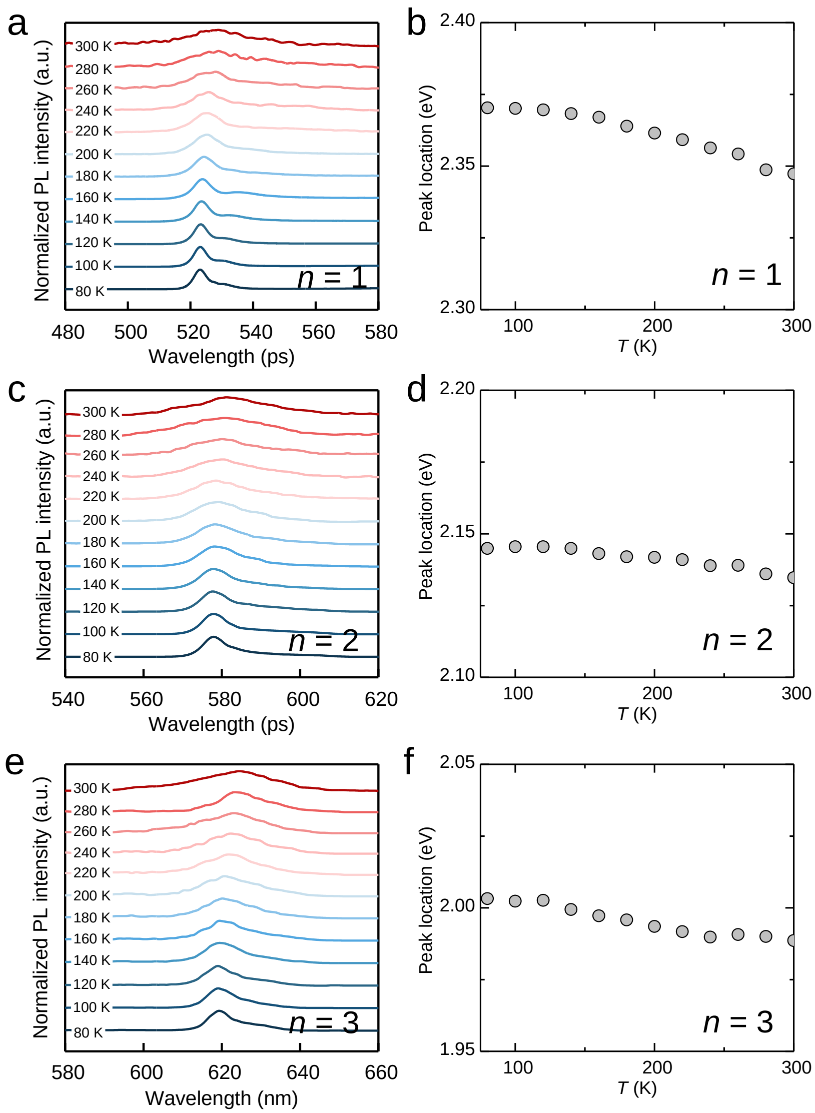

Fig. S22. Temperature-resolved photoluminescence spectra of (a) $n = 1$, (c) $n = 2$, and (e) $n = 3$. The temperature range is from 80 K to 300 K with steps of 20 K. Position of the exciton peak of (b) $n = 1$, (d) $n = 2$, and (f) $n = 3$ as a function of temperature.

Fig. S23

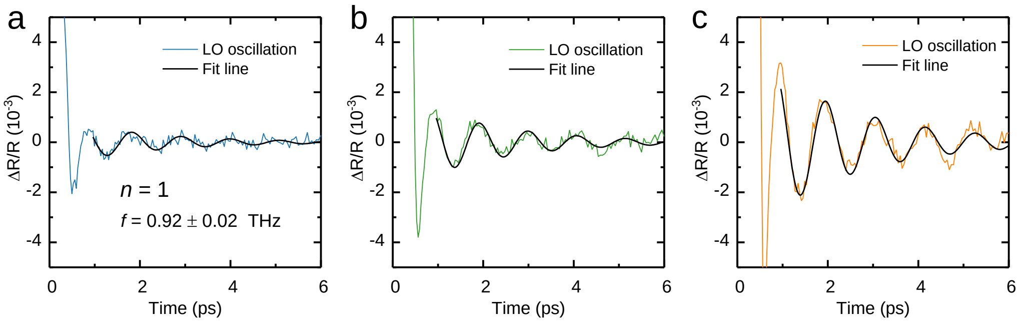

Fig. S23. Coherent optical phonon propagation in (4AMP)PbI_4 sample.

Fig. S24

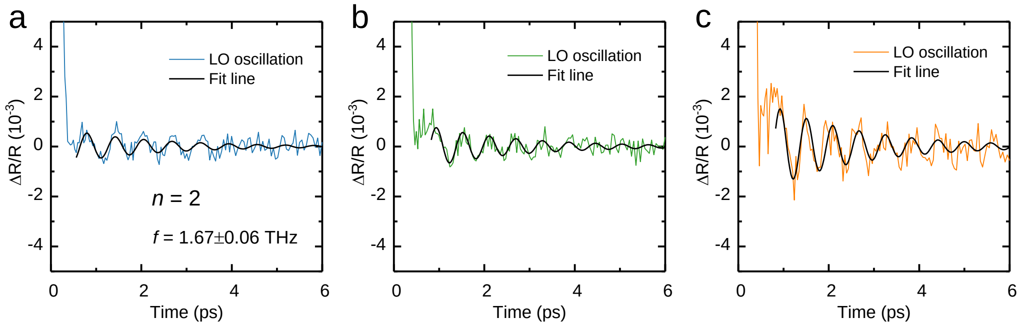

Fig. S24. Coherent optical phonon propagation in $(4AMP)Pb_2I_7$ sample.

Fig. S25

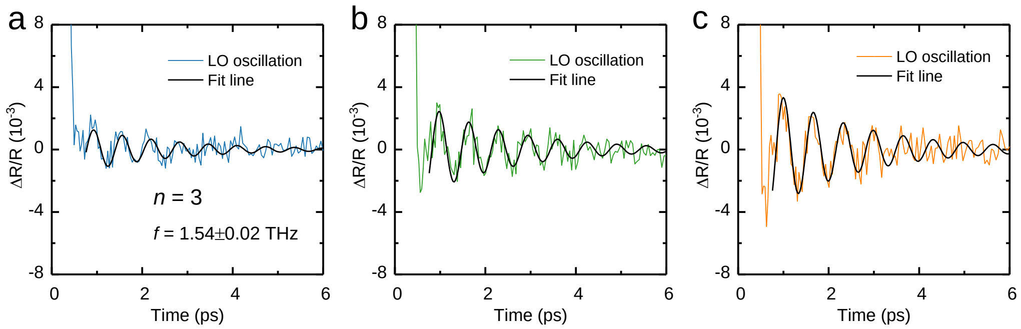

Fig. S25. Coherent optical phonon propagation in $(4AMP)Pb_3I_{10}$ sample.

Fig. S26

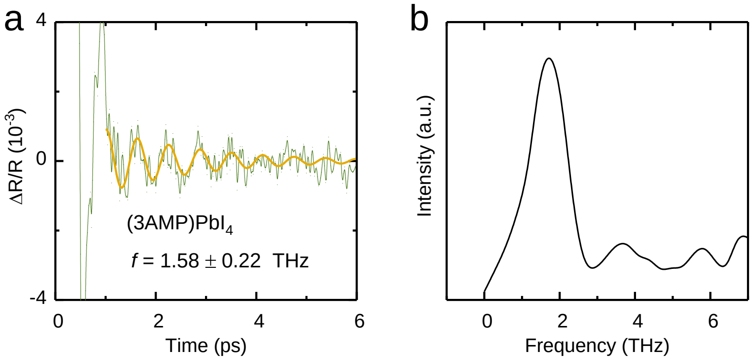

Fig. S26. (a) Coherent optical phonon propagation in $(3AMP)PbI_4$ sample. (b) Normalized Fourier transform amplitude of the coherent optical phonon oscillations.

Fig. S27

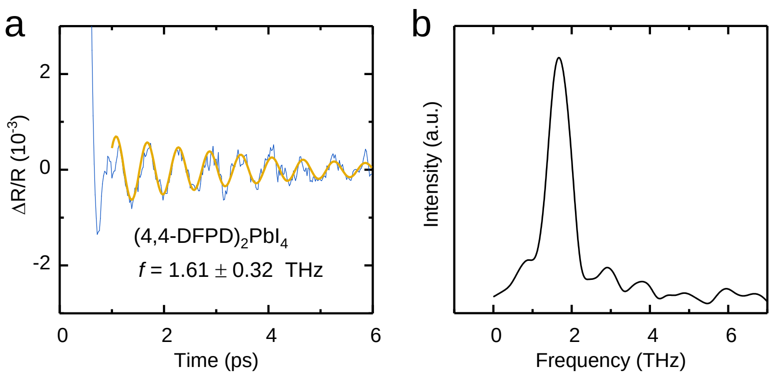

Fig. S27. (a) Coherent optical phonon propagation in (4,4-DFPD)$_2$PbI$_4$ sample. (b) Normalized Fourier transform amplitude of the coherent optical phonon oscillations.

Fig. S28

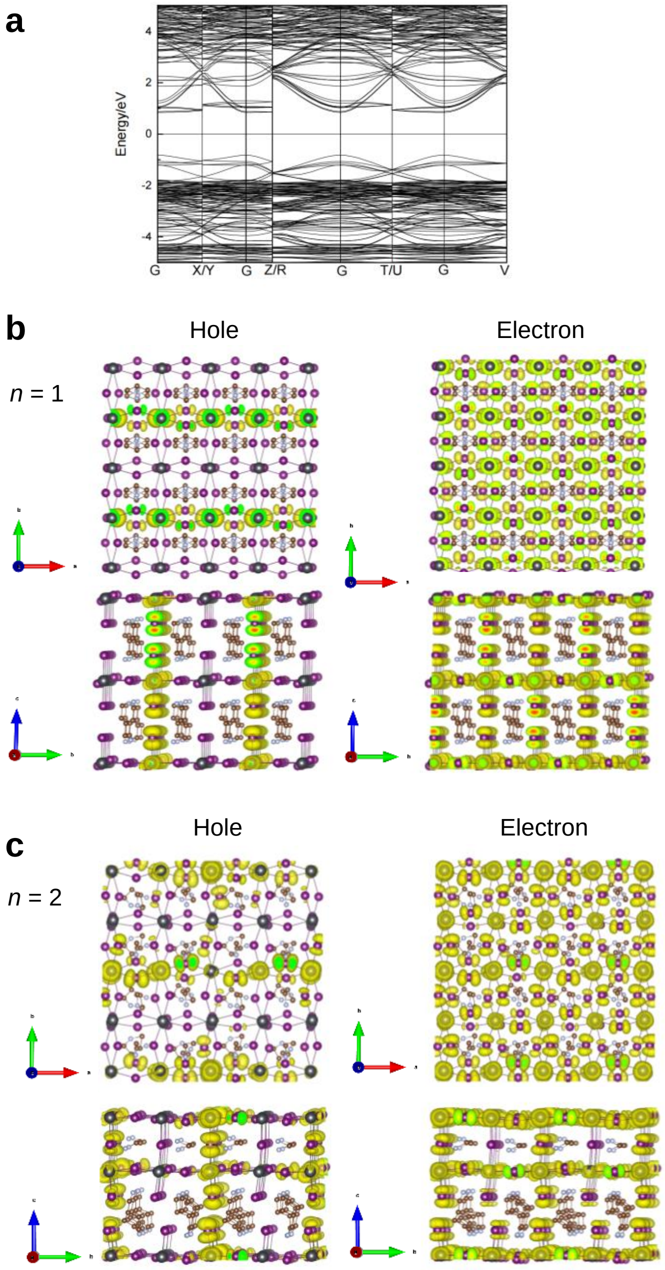

Fig. S28. (a) Electronic band structures of (4AMP)PbI_4. (b) Hole and electron distribution in *n* = 1 samples. (c) Hole and electron in *n* = 2 samples.

Fig. S29

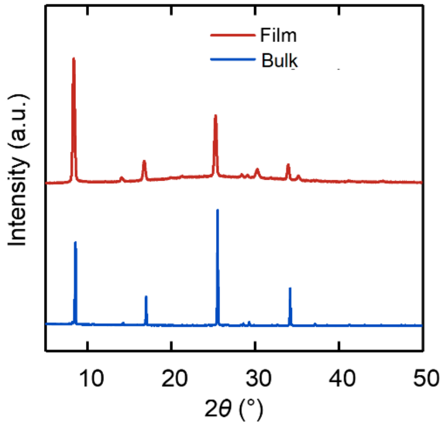

Fig. S29. XRD patterns of $(4AMP)PbI_4$: comparison between bulk and thin film specimens.

Fig. S30

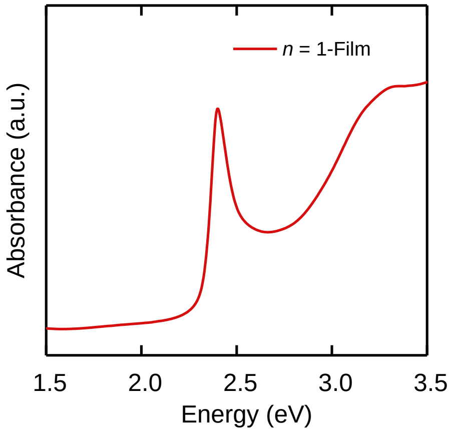

Fig. S30. Optical absorption spectrum of (4AMP)PbI_4 thin film.

Fig. S31

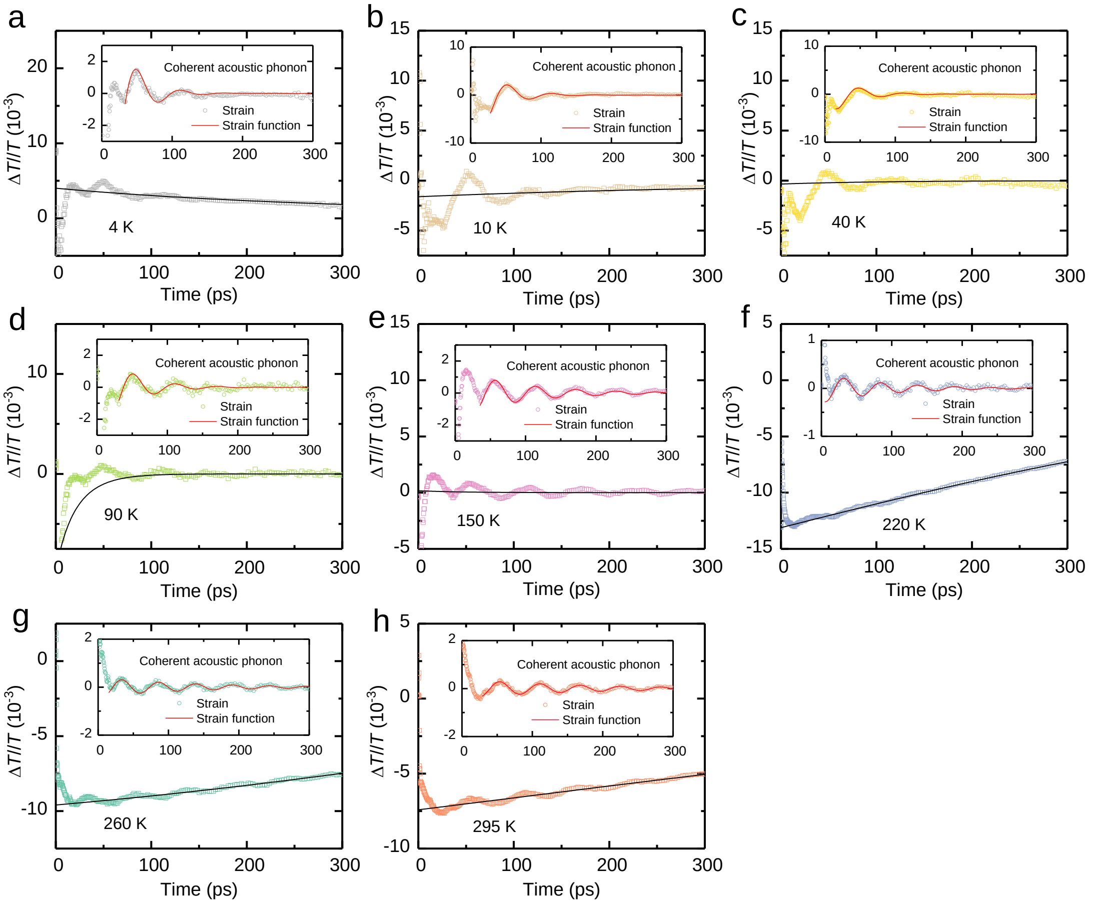

Fig. S31. (a-h) TA kinetics signals of $(4AMP)PbI_4$ thin film at pump wavelength of 500 nm in the temperature range 4-295 K with a solid black line according to ABC recombination model. The Coherent acoustic phonon oscillation after subtracting contributions from carrier recombination is shown in each Figure.

Fig. S32

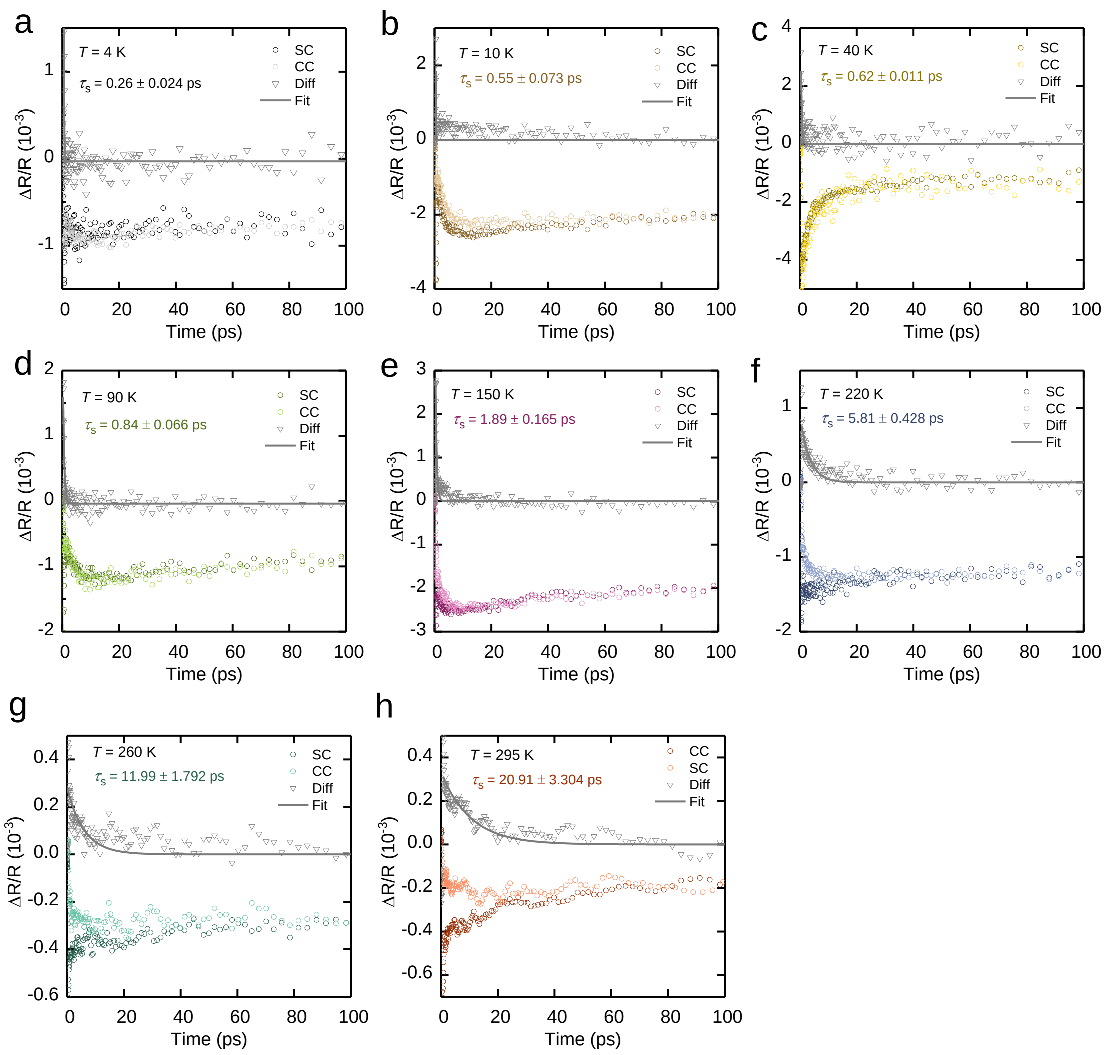

Fig. S32. (a-h) SC and CC pump-probe induced spin dynamics of $(4AMP)PbI_4$ thin film as a function time delay, in the temperature range 4-295 K.

Fig. S33

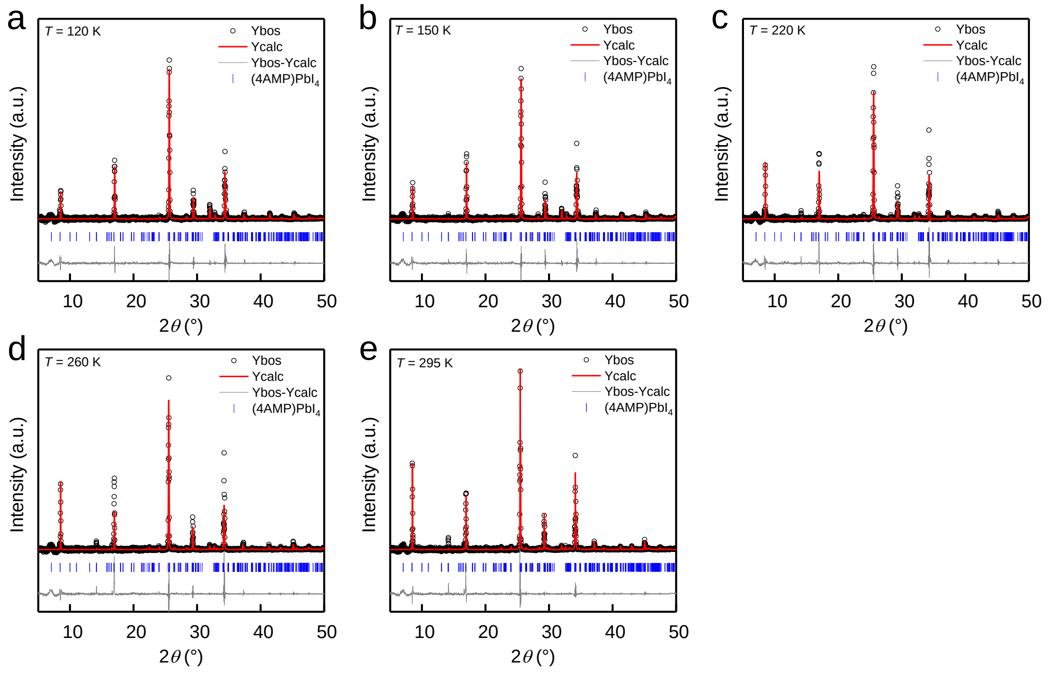

Fig. S33. XRD Patterns and Rietveld refinements of (4AMP)PbI_4 powder sample at representative temperature of (a) 120 K; (b) 150 K; (c) 220 K; (d) 260 K and (e) 250 K.

SI References

1. D. Campi, C. Coriasso, Optical nonlinearities in multiple quantum wells: Generalized Elliott formula. *Phys. Rev. B* **51**, 10719–10728 (1995).
2. X. Chen *et al.*, Tuning Spin-Polarized Lifetime in Two-Dimensional Metal-Halide Perovskite through Exciton Binding Energy. *J. Am. Chem. Soc.* **143**, 19438–19445 (2021).
3. W. Tao, Q. Zhou, H. Zhu, Dynamic polaronic screening for anomalous exciton spin relaxation in two-dimensional lead halide perovskites. *Sci. Adv.* **6**, 2–10 (2020).
4. X. Chen *et al.*, Impact of Layer Thickness on the Charge Carrier and Spin Coherence Lifetime in Two-Dimensional Layered Perovskite Single Crystals. *ACS Energy Lett.* **3**, 2273–2279 (2018).
5. H. A. Odelola, J. Koza, Spintronics: Fundamentals and applications. *Rev. Mod. Phys.* **76**, 323–410 (2004).
6. S. Gong *et al.*, Ultrafast dynamics in perovskite-based optoelectronic devices. *Cell Reports Phys. Sci.* **4**, 101580 (2023).
7. P. Ruello, V. E. Gusev, Physical mechanisms of coherent acoustic phonons generation by ultrafast laser action. *Ultrasonics* **56**, 21–35 (2015).
8. P. A. Mante, C. C. Stoumpos, M. G. Kanatzidis, A. Yartsev, Electron-acoustic phonon coupling in single crystal $CH_3NH_3PbI_3$ perovskites revealed by coherent acoustic phonons. *Nat. Commun.* **8**, 14398 (2017).
9. J. Fu *et al.*, Strain propagation in layered two-dimensional halide perovskites. *Sci. Adv.* **8**, eabq1971 (2022).
10. J. Bhosale *et al.*, Temperature dependence of band gaps in semiconductors: Electron-phonon interaction. *Phys. Rev. B* **86**, 195208 (2012).
11. W. Paritmongkol, E. R. Powers, N. S. Dahod, W. A. Tisdale, Two Origins of Broadband Emission in Multilayered 2D Lead Iodide Perovskites. *J. Phys. Chem. Lett.* **11**, 8565–8572 (2020).
12. M. Gramlich, C. Lampe, J. Drewniok, A. S. Urban, How Exciton-Phonon Coupling Impacts Photoluminescence in Halide Perovskite Nanoplatelets. *J. Phys. Chem. Lett.* **12**, 11371–11377 (2021).
